# Physiologically Based Pharmacokinetic Modeling to Describe the CYP2D6 Activity Score-Dependent Metabolism of Paroxetine, Atomoxetine and Risperidone

**DOI:** 10.3390/pharmaceutics14081734

**Published:** 2022-08-18

**Authors:** Simeon Rüdesheim, Dominik Selzer, Thomas Mürdter, Svitlana Igel, Reinhold Kerb, Matthias Schwab, Thorsten Lehr

**Affiliations:** 1 Department of Clinical Pharmacy, Saarland University, 66123 Saarbrücken, Germany; 2 Dr. Margarete Fischer-Bosch-Institute of Clinical Pharmacology, 70376 Stuttgart, Germany; 3 Departments of Clinical Pharmacology, Pharmacy and Biochemistry, University of Tübingen, 72076 Tübingen, Germany; 4 Cluster of Excellence iFIT (EXC2180) “Image-Guided and Functionally Instructed Tumor Therapies”, University of Tübingen, 72076 Tübingen, Germany

**Keywords:** physiologically based pharmacokinetic (PBPK) modeling, paroxetine, atomoxetine, risperidone, cytochrome P450 2D6 (CYP2D6)

## Abstract

The cytochrome P450 2D6 (*CYP2D6*) genotype is the single most important determinant of CYP2D6 activity as well as interindividual and interpopulation variability in CYP2D6 activity. Here, the CYP2D6 activity score provides an established tool to categorize the large number of CYP2D6 alleles by activity and facilitates the process of genotype-to-phenotype translation. Compared to the broad traditional phenotype categories, the CYP2D6 activity score additionally serves as a superior scale of CYP2D6 activity due to its finer graduation. Physiologically based pharmacokinetic (PBPK) models have been successfully used to describe and predict the activity score-dependent metabolism of CYP2D6 substrates. This study aimed to describe CYP2D6 drug–gene interactions (DGIs) of important CYP2D6 substrates paroxetine, atomoxetine and risperidone by developing a substrate-independent approach to model their activity score-dependent metabolism. The models were developed in PK-Sim^®^, using a total of 57 plasma concentration–time profiles, and showed good performance, especially in DGI scenarios where 10/12, 5/5 and 7/7 of DGI AUC_last_ ratios and 9/12, 5/5 and 7/7 of DGI C_max_ ratios were within the prediction success limits. Finally, the models were used to predict their compound’s exposure for different CYP2D6 activity scores during steady state. Here, predicted DGI AUC_ss_ ratios were 3.4, 13.6 and 2.0 (poor metabolizers; activity score = 0) and 0.2, 0.5 and 0.95 (ultrarapid metabolizers; activity score = 3) for paroxetine, atomoxetine and risperidone active moiety (risperidone + 9-hydroxyrisperidone), respectively.

## 1. Introduction

Differences in CYP2D6 activity have been described as early as the 1970s [1,2] and have since been a major focus of clinical research, as CYP2D6 is involved in the metabolism of approximately 20% of clinically relevant drugs [3]. Polymorphic expression of the *CYP2D6* gene has been identified as the single most important determinant of CYP2D6 activity leading to a substantial interindividual and interpopulation variability observed in the pharmacokinetics of CYP2D6 substrates [4]. For instance, homozygous carriers of loss-of-function alleles (genetic poor metabolizers) show no detectable CYP2D6 activity [3] and are consequently unable to biotransform drugs via CYP2D6 [4]. In contrast, individuals carrying multiple copies of a normal function allele (genetic ultrarapid metabolizers) generally display increased CYP2D6 activity compared to homozygous carriers of wildtype alleles (genetic normal metabolizers) [5] and show accelerated biotransformation of CYP2D6 substrates. Both poor and ultrarapid metabolizers are at an increased risk for experiencing dose-dependent adverse drug effects or a lack of response, depending on the CYP2D6 substrate [4].

To address this issue, pharmacogenetic testing for variants in *CYP2D6* has become an important cornerstone for personalized drug therapy [6] with the overall aim to improve efficacy and patient safety while simultaneously reducing costs of drug therapy, for example, due to hospitalizations caused by adverse drug reactions (ADRs) [7]. Here, the CYP2D6 activity score system serves as an indispensable tool to translate genotype data into phenotypes. Moreover, the activity score system can provide more fine-grained estimations of CYP2D6-dependent drug clearance [8], and, by extension, serves as an important basis for the development of actionable clinical guidelines [9]. Its main benefit is the aggregation of the >10,000 possible *CYP2D6* [10,11,12,13] genotypes into a manageable scoring system by assigning a numeric value ranging from 0 to 1 to *CYP2D6* alleles based on their in vitro and in vivo CYP2D6 activity [8]. Based on their genetic makeup, an individual’s activity score can subsequently be translated into one of the following metabolizer phenotypes: poor (AS = 0), intermediate (0 < AS ≤ 1), normal (1 < AS ≤ 2.25) or ultrarapid metabolizer (AS > 2.25) [5]. Importantly, these phenotype categories are not identical to the “traditional” phenotype definitions, determined using phenotyping methods (e.g., calculating urinary metabolic ratios or screening for null alleles [8,14,15]). Consequently, the “traditional” extensive metabolizer and the normal metabolizer categories only display a limited intersection in terms of CYP2D6 activity [16].

While the activity score system’s main purpose is the facilitation of genotype-to-phenotype translation, it has been suggested to provide an even finer graduated scale of CYP2D6 activity, allowing to infer a percentage of CYP2D6 activity (relative to activity score = 2) compared to the broad categories of traditional phenotypes [5]. Findings obtained from previously published physiologically based pharmacokinetic (PBPK) models of important CYP2D6 substrates dextromethorphan and metoprolol demonstrated a possibility to translate the CYP2D6 activity score into an apparent CYP2D6 clearance, reflected in increasing CYP2D6 catalytic rate constant (k_cat_) values with increasing activity scores [15,17]. Here, drug–gene interaction (DGI) PBPK models provide a practical approach to mechanistically implement the activity score-dependent metabolism of CYP2D6 substrates [18].

The objective of this study was to implement the activity score-dependent metabolism in PBPK models of various important CYP2D6 substrates. For this, new models were developed for the selective serotonin reuptake inhibitor (SSRI) and CYP2D6 inhibitor paroxetine and the norepinephrine reuptake inhibitor (NRI) atomoxetine. The continuous scale of activity score-dependent metabolism derived from PBPK models of CYP2D6 substrates dextromethorphan and metoprolol was additionally implemented in these new PBPK models as well as an established PBPK model of the atypical antipsychotic risperidone, originally based on traditional phenotype categories.

## 2. Materials and Methods

### 2.1. Workflow

**Figure 1:**
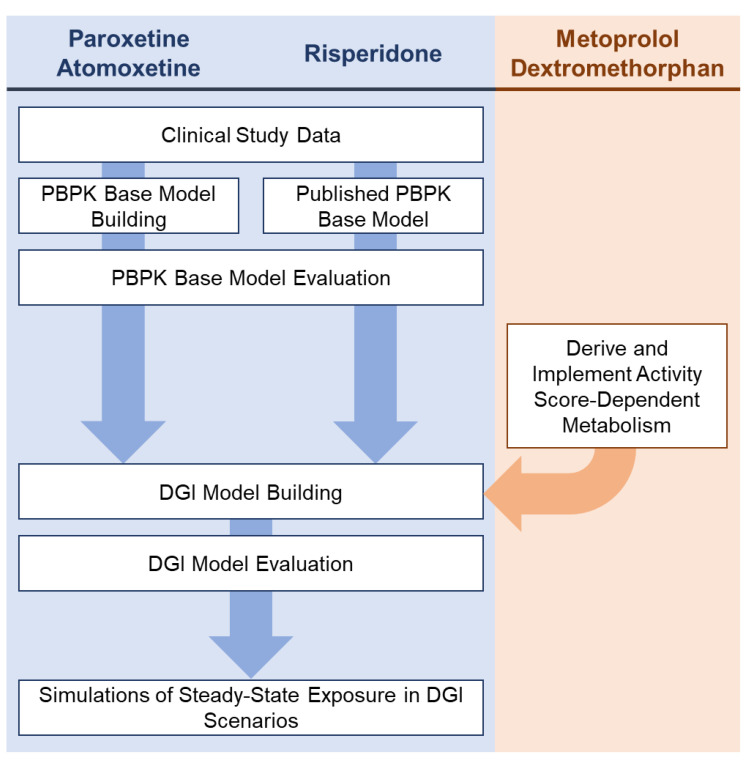
Workflow of the literature search of clinical study data, PBPK base model building, PBPK base model evaluation, DGI model building, DGI model evaluation and DGI model application processes for the modeled compounds.

The overall workflow for this study included (I) the collection of clinical study data, (II) PBPK base model building (paroxetine, atomoxetine) and (III) PBPK base model evaluation (paroxetine, atomoxetine. Published PBPK DGI models (metoprolol, dextromethorphan) were used to (IV) derive the scale of their CYP2D6 activity score-dependent metabolism and implement it during the (V) DGI model building process (paroxetine, atomoxetine, and risperidone). After (VI) DGI model evaluation (paroxetine, atomoxetine, and risperidone), the models were applied to (VII) simulate steady-state exposure in different DGI scenarios (paroxetine, atomoxetine, and risperidone). Figure 1 schematically depicts the workflow for this study.

### 2.2. Software

PBPK models were developed in PK-Sim^®^ (Open Systems Pharmacology Suite 10, www.open-systems-pharmacology.org, 2021). Clinical study data from the literature were digitized with GetData Graph Digitizer 2.26.0.20 (© S. Fedorov, http://www.getdata-graph-digitizer.com/index.php, 2013) according to best practices [19]. Sensitivity analyses and model parameter optimizations (Monte Carlo algorithm) were performed within PK-Sim^®^. Pharmacokinetic parameters, model performance metrics and plots were calculated and generated using Python (version 3.10.4, Python Software Foundation, Wilmington, DE, USA, 2022). Regression analyses were performed using ordinary least squares (OLS) regression utilizing the *statsmodels* package (version 0.13.2, https://github.com/statsmodels/statsmodels, 2021) [20].

### 2.3. Clinical Study Data

An extensive literature search was conducted to gather individual and aggregated plasma concentration–time profiles after intravenous and oral administrations in single and multiple-dose regimes of paroxetine, atomoxetine, and risperidone. Additionally, population or individual demographics (sex, age, weight, and height) alongside CYP2D6 activity (phenotype, genotype, or activity score) were extracted from the respective studies. The collected plasma concentration–time profiles were split into a training dataset for model development, and a test dataset for model performance evaluation. Studies for model training were selected to include sufficient data covering different routes of administration (intravenous and oral), formulations (oral solution or solid dosage forms), a wide range of doses as well different *CYP2D6* genotypes, activity scores or phenotypes.

### 2.4. PBPK Base Model Building

The paroxetine and atomoxetine PBPK base models were developed using a sequential approach. First, appropriate quantitative structure–activity relationship (QSAR) methods to estimate partition coefficients and cellular permeabilities were selected by the smallest residual error for fitting simulations of intravenous administrations (paroxetine) or all studies of the training dataset (atomoxetine) to their observed data. Second, simulations of administrations of oral solutions were optimized against the respective clinical data to estimate intestinal permeability. Third, parameters for CYP2D6-independent metabolism were informed by fitting simulations of single and multiple-dose oral administrations in poor metabolizers of CYP2D6 to their respective observed data. Finally, parameters for CYP2D6-mediated metabolism were optimized for studies of the training dataset where the volunteers were extensive metabolizers. Here, the term “extensive metabolizers” was used to group populations that were either phenotyped via traditional phenotyping methods or populations, which were not phenotyped.

For the risperidone base model, a published PBPK model by Kneller et al. [21] was used.

### 2.5. PBPK Base Model Evaluation

The performance of the presented models was evaluated using graphical and statistical methods. First, predicted plasma concentration–time profiles were compared graphically with measured data from the respective clinical studies by plotting model population predictions (arithmetic mean ± SD) together with observed data points. For this purpose, virtual populations of 1000 individuals were created based on the population characteristics reported in the respective publication. System-dependent parameters, such as age, body weight, height, organ weights, blood flow rates, and tissue composition, were varied by the implemented algorithm in PK-Sim^®^. Second, the plasma concentration values of all studies using the predicted arithmetic mean of the population were plotted against the corresponding observed values in goodness-of-fit plots. In addition, model performance was evaluated by a comparison of predicted to observed area under the concentration–time curve (AUC) and maximum plasma concentration (C_max_) values. All AUC values (predicted as well as observed) were calculated from the time of the first concentration measurement to the time of the last concentration measurement (AUC_last_).

Finally, as quantitative measures of the model performance, the mean relative deviation (MRD) of all predicted plasma concentrations (Equation (1)) and the geometric mean fold error (GMFE) of all predicted AUC_last_ and C_max_ values (Equation (2)) were calculated.

(1) $${\mathrm{MRD}=10}^{x};\;x=\sqrt{\frac{\sum_{i=1}^{k}{\;(\log}_{10}\hat{c_{i}}{\;-\;\log}_{10}c_{i}{)}^{2}}{k}}$$

where $\hat{c_{i}}$ = predicted plasma concentration that corresponds to the i-th observed concentration, $c_{i}$ = i-th observed plasma concentration, and k = number of observed values.

(2) $${\mathrm{GMFE}=10}^{x};\;x=\frac{\sum_{i=1}^{m}\;\left|\log_{10}\;\left(\frac{\hat{\rho_{i}}}{\rho_{i}}\right)\right|\;}{m}$$

where $\hat{P_{i}}$ = predicted AUC_last_ or C_max_ value of study, $p_{i}$ = corresponding observed AUC_last_ or C_max_ value of study, i, and m = total number of studies.

### 2.6. Local Sensitivity Analysis

Local sensitivity of the AUC_0–24 h_ of paroxetine, atomoxetine, risperidone or 9-hydroxyrisperidone to single parameter changes was analyzed for simulations of single orally administered standard doses of paroxetine, atomoxetine, and risperidone, respectively. Parameters were included in the analysis if they have been optimized (intestinal permeabilities and k_cat_ values), if they are associated with optimized parameters (K_M_ values) or if they might have a strong impact due to calculation and QSAR methods used (lipophilicities, pK_a_ values and fractions unbound (f_u_)). A detailed description of the model sensitivity analysis is provided in Section S1.4 of Supplementary Materials S1. Overviews of all varied parameters for the respective compounds are provided in Sections S2.2.3, S3.2.3 and S4.2.3 of Supplementary Materials S1.

### 2.7. DGI Model Building

CYP2D6-dependent clearance processes were modeled using Michaelis–Menten kinetics according to Equation (3):

(3) $$v=\frac{V_{\max}\;\cdot \;S}{K_{M}+S}=\frac{k_{\mathrm{cat}\;}\cdot \;E\;\cdot \;S}{K_{M}+S}$$

where v = reaction velocity at substrate concentration S, V_max_ = maximum reaction velocity, K_M_ = Michaelis–Menten constant, k_cat_ = catalytic rate constant, and E = enzyme concentration.

The CYP2D6 DGI models for paroxetine, atomoxetine, and risperidone were developed based on two previously published models for the CYP2D6 substrates metoprolol [17] and dextromethorphan [15]. Relative k_cat_ values, defined as the ratio of k_cat_ values for populations with a variant activity score and the k_cat_ for populations with an activity score of 2 (corresponding to 100% of CYP2D6 activity), were calculated according to Equation (4):

(4) $$k_{\mathrm{cat},\;\mathrm{rel},\;\mathrm{AS}=i}=\frac{k_{\mathrm{cat},\;\mathrm{AS}=i}}{k_{\mathrm{cat},\;\mathrm{AS}=2}}\times \;100\%$$

where k_cat, rel, AS = i_ = k_cat_ for the investigated activity score relative to AS = 2 and k_cat, AS = i_ = k_cat_ for activity score i.

Activity scores were assigned according to the current consensus [5]. CYP2D6 k_cat, rel_ values used to describe the activity score-dependent metabolism of metoprolol [17] and dextromethorphan [15] in their respective DGI PBPK models, were analyzed using OLS regression (polynomial of degree 2, no intercept). For the paroxetine, atomoxetine and risperidone models, CYP2D6 k_cat_ values were optimized for studies, which reported plasma concentration–time profiles of populations with two wildtype alleles (AS = 2) and were set to 0 for poor metabolizers of CYP2D6 (AS = 0) as they were assumed to show no CYP2D6 activity [3]. Subsequently, k_cat_ values for all other modeled activity scores were calculated using the polynomial equation obtained from the OLS regression of metoprolol and dextromethorphan k_cat_ values. Here, CYP2D6 K_M_ values as well as CYP2D6 reference concentrations were kept constant over the whole range of modeled activity scores.

### 2.8. DGI Model Evaluation

To evaluate the performance of the presented DGI models, as well as the implemented scale of CYP2D6 activity score-dependent metabolism derived from the published metoprolol and dextromethorphan PBPK DGI models, predicted plasma concentration–time profiles were plotted alongside their respective observed data. Plasma concentration–time profiles for populations with variant activity scores were compared to profiles of a population with normal activity (AS = 2) in studies reporting activity scores or genotypes, whereas plasma concentration–time profiles for variant phenotypes were compared to those of the extensive metabolizer phenotype, where only CYP2D6 phenotypes were reported. Furthermore, predicted DGI PK ratios (AUC_last_ and C_max_ ratios) (Equation (5)) were evaluated for study populations with variant CYP2D6 activity scores or phenotypes alongside GMFE values (Equation (2)) for the predicted PK ratios.

(5) $$\mathrm{DGI}\;\mathrm{PK}\;\mathrm{ratio}=\;\frac{{\mathrm{PK}}_{\mathrm{DGI}}\;}{{\mathrm{PK}}_{\mathrm{reference}}\;}$$

where PK_DGI_ = AUC_last_ or C_max_ of either a variant activity score or a variant phenotype; PK_reference_ = AUC_last_ or C_max_ of either AS = 2 or the extensive metabolizer phenotype, respectively.

Additionally, steady-state exposures (AUC_ss_) of model compounds were predicted for different CYP2D6 activity scores. Here, simulations were performed for individuals with different activity scores after multiple oral doses of 40 mg paroxetine, 40 mg atomoxetine, or 2 mg risperidone.

## 3. Results

### 3.1. PBPK Base Model Building

A total of 57 plasma concentration–time profiles obtained from 29 published clinical trials were used for the development and the evaluation of the paroxetine, atomoxetine, and risperidone PBPK models and are summarized in Table 1. Clinical study tables providing comprehensive information such as individual and population demographics (sex, age, weight, and height) and CYP2D6 activity (phenotype, genotype, or activity score) as well as the assignment of the study to the respective dataset are presented in Sections S2.1.2, S3.1.2 and S4.1.2 of Supplementary Materials S1 for paroxetine, atomoxetine, and risperidone, respectively.

**Figure 2:**
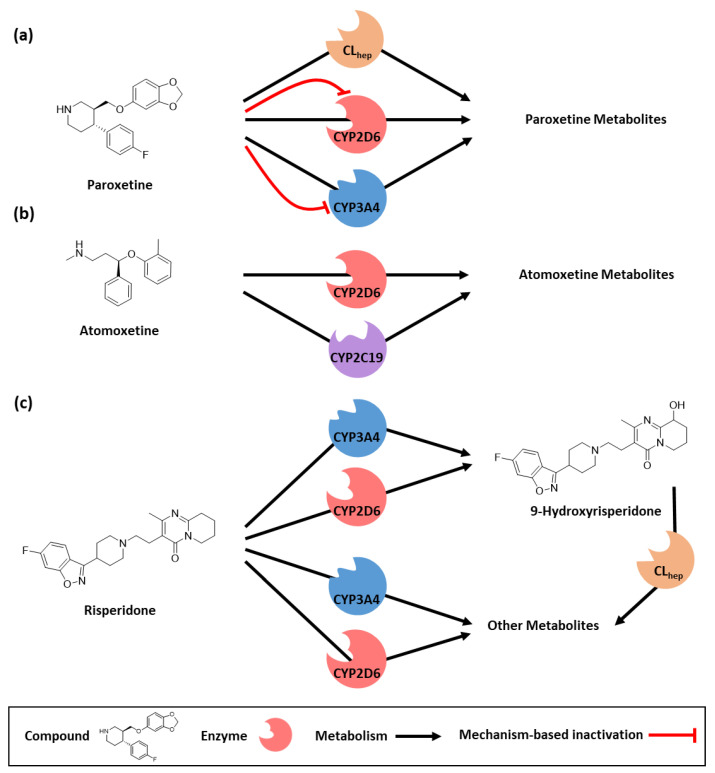
Implemented metabolic pathways for the modeled compounds. (**a**) Paroxetine is metabolized via CYP2D6, CYP3A4, and an unspecific clearance process [22]. Moreover, paroxetine is a mechanism-based inhibitor of CYP2D6 and CYP3A4 resulting in an irreversible auto-inhibition of paroxetine metabolism [23,55]. Paroxetine metabolites were not included as model compounds. (**b**) Atomoxetine is metabolized via CYP2D6 and CYP2C19 [24]. Atomoxetine metabolites were not included as model compounds. (**c**) Risperidone is metabolized to its active metabolite 9-hydroxyrisperidone via CYP2D6 and CYP3A4 [56]. Moreover, other metabolites are formed via CYP2D6- and CYP3A4-mediated metabolism [56]. 9-Hydroxyrisperidone is metabolized via an unspecific hepatic clearance process. Other risperidone metabolites were not included in the model. CL_hep_: unspecific hepatic clearance, and CYP: cytochrome P450.

For the paroxetine PBPK model, a total of 33 plasma concentration–time profiles where paroxetine was administered as an intravenous infusion (four profiles) or orally in single (16 profiles) or multiple (13 profiles) doses were used to develop the paroxetine PBPK model. Here, administered doses ranged from 10 to 70 mg of paroxetine. The paroxetine PBPK model incorporates CYP2D6- and CYP3A4-dependent metabolism of paroxetine [22] as well as irreversible inhibition of CYP2D6 and CYP3A4 [23]. Additionally, an unspecific hepatic clearance process and renal elimination via passive glomerular filtration were included. A schematic overview of implemented paroxetine metabolic pathways is provided in Figure 2a. Drug-dependent model parameters for paroxetine are presented in Section S2.1.1 of Supplementary Materials S1.

The atomoxetine PBPK model was developed using 12 plasma concentration–time profiles after oral administrations of atomoxetine in single (nine profiles) and multiple-dose administrations (three profiles) with doses of administered atomoxetine ranging between 20 and 50 mg. The atomoxetine PBPK model includes metabolism via CYP2D6 and CYP2C19 [24] as well as a passive glomerular filtration process. Figure 2b depicts the pathways implemented in the atomoxetine model. Atomoxetine drug-dependent model parameters are presented in Section S3.1.1 of Supplementary Materials S1.

An overview of risperidone model pathways as published by Kneller et al. [21] is provided in Figure 2c. Risperidone and 9-hydroxyrisperidone drug-dependent parameters are listed in Section S4.1.1 of Supplementary Materials S1.

**Table 1:** Summary of clinical studies used for model development and evaluation.

Study	Dose [mg]	*n*	Females [%]	Age [Years]	Weight [kg]		CYP2D6 Status	References
*** Paroxetine ***								
Belle et al., 2002	20, p.o. (tab)	22	23	38 (20–49)	-		EM	[25]
Calvo et al., 2004	20, p.o. (tab)	25	64	26	64		-	[26]
Chen et al., 2015	25, p.o. (cr)	24	42	26 (18–45)	61		0.5, 1.0, 1.5, 2	[27]
Lund et al., 1982	23–28, i.v. (inf.); 45, p.o. (sol)	4	0	(24–28)	(66–88)		-	[28]
Massaroti et al., 2005	20, p.o. (tab)	28	0	28 (18–42)	72 (57–87)		-	[29]
McClelland et al., 1984	70, p.o. (tab)	28	0	31 (22–44)	-		-	[30]
Mürdter et al., 2016	40, p.o. (tab)	16	100	26 (21–43)	61 (48–74)		0, 0.5, 0.75, 1, 2, 3	[31,32,33]
Schoedel et al., 2012	20, p.o. (tab)	14	14	34 (19–55)	75		-	[34]
Segura et al., 2005	20, p.o. (tab)	7	0	23	65		EM	[35]
Sindrup et al., 1992	40, p.o. (tab)	17	0	25 (20–39)	77 (65–95)		EM, PM	[36]
van der Lee et al., 2007	20, p.o. (tab)	26	69	44 (18–64)	69 (51–89)		EM	[37]
Yasui-Furukori et al., 2006	20, p.o. (tab)	12	25	25 (20–35)	58 (46–75)		1.25	[38]
Yasui-Furukori et al., 2007	20, p.o. (tab)	13	23	24 (21–35)	57 (45–67)		EM	[39]
Yoon et al., 2000	40, p.o. (tab)	16	13	22	64		0, 0.5, 1.25, 2	[40]
***Atomoxetine***								
Belle et al., 2002	20, p.o. (tab)	22	23	38 (20–49)	-		EM	[25]
Byeon et al., 2015	40, p.o. (tab)	62	0	23	66		0, 1.25, 2	[41]
Cui et al., 2007	40-80, p.o. (tab)	16	33	(20–29)	(53–72)		1	[42]
Kim et al., 2018	20, p.o. (tab)	19	0	(19–25)	(49–73)		0.5, 2	[43]
Nakano et al., 2016	50, p.o. (tab, sol)	42	0	23 (20–37)	62 (52–76)		EM	[44]
Sauer et al., 2003	20, p.o. (tab)	7	0	41 (19–54)	-		EM, PM	[45]
Todor et al., 2016	40, p.o. (tab)	30	0	(18–55)	-		EM, PM	[46]
***Risperidone***								
Bondolfi et al., 2001	2, p.o. (tab)	11	27	43 (18–63)	-		EM, PM	[47]
Darwish et al., 2015	2, p.o. (tab)	36	33	32	79		-	[48]
Kim et al., 2008	1, p.o. (tab)	10	0	(23–38)	(65–80)		1.25	[49]
Markowitz et al., 2002	1, p.o. (tab)	11	21	28 (22–42)	-		-	[50]
Mahatthanatrakul 2007	4, p.o. (tab)	10	0	31	(55–76)		-	[51]
Mahatthanatrakul 2012	2, p.o. (tab)	10	0	33 (23–44)	64 (55–76)		-	[52]
Nakagami et al., 2005	1, p.o. (tab)	12	0	24 (20–28)	65 (53–86)		1	[53]
Novalbos et al., 2010	1, p.o. (tab)	71	51	23 (19–34)	66 (43–106)		0, 1, 2, 3	[21,54]

Demographic parameters are given as the mean (range). CYP2D6 status is reported as the mode of the study population phenotype or activity score or the different phenotypes and activity scores reported for the respective study sub-populations. -: not given, cr: controlled release tablet, EM: extensive metabolizer, inf: infusion, i.v. intravenous, PM: poor metabolizer, p.o.: oral, sol: oral solution, and tab: tablet.

### 3.2. PBPK Base Model Evaluation

**Figure 3:**
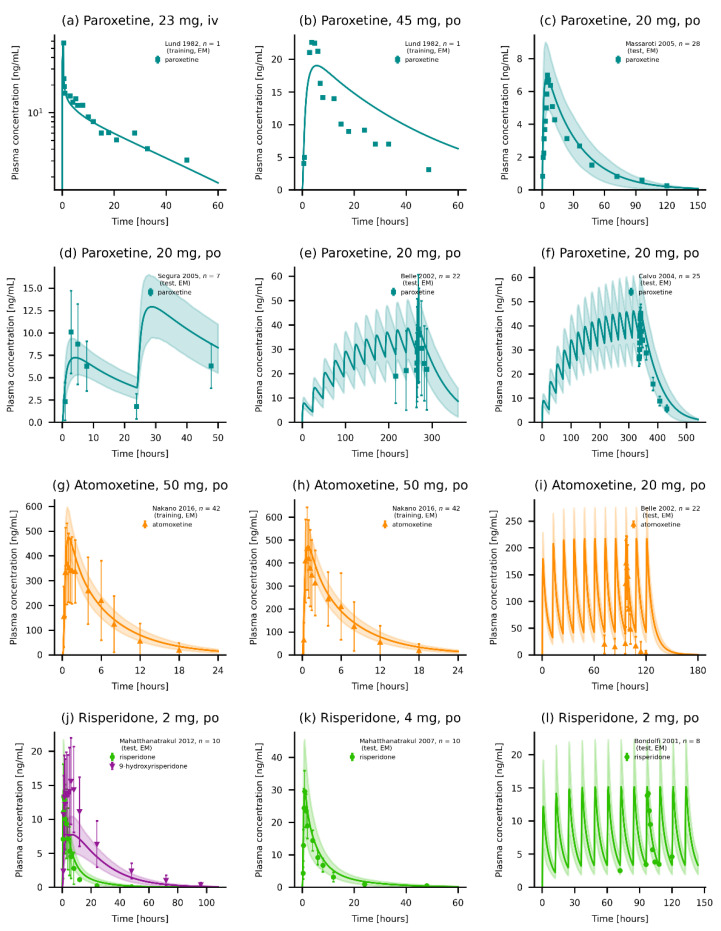
Plasma concentrations of the modeled compounds. (**a**–**f**) Model predictions of paroxetine of selected (**a**–**c**) single-dose administrations of (**a**) an intravenous infusion, (**b**) an oral solution, and (**c**) a normal release tablet. (**d**–**f**) Multiple-dose administrations of paroxetine as normal release tablets [25,26,28,29,35]. (**g**–**i**) Model predictions of atomoxetine as (**g**,**h**) single-dose administrations of (**g**) an oral solution, (**h**) a capsule and (**i**) multiple-dose administration of atomoxetine [25,44]. (**j**–**l**) Model predictions of risperidone and 9-hydroxyrisperidone (if available) of (**j**,**k**) single-dose administrations and (**l**) a multiple-dose administration of risperidone [47,51,52]. Individual predictions are shown as lines. Population predictions (*n* = 1000) are shown as lines with ribbons (arithmetic mean ± standard deviation (SD)), and symbols present the corresponding observed data ± SD (if available). Detailed information on all clinical studies is listed in Section S2.1.2 [27,30,33,34,36,37,38,39,40], Section S3.1.2 [41,42,43,45,57] and Section S4.1.2 [48,49,53,54] of the Supplementary Materials S1. iv: intravenous, and po: oral.

The three presented models could accurately predict the plasma concentrations for their respective model compounds. A representative selection of plots displaying predicted compared to observed plasma concentration–time profiles for paroxetine (a–f), atomoxetine (g–i) and risperidone and its metabolite 9-hydroxrisperidone (j–l) is shown in Figure 3.

Additionally, plots displaying predicted compared to observed plasma concentration–time profiles of the three compounds alongside their respective GMFEs for AUC_last_ and C_max_ values as well as MRD of predicted and observed plasma concentrations are given in Sections S2.2.1, S3.2.1, and S4.2.1 of Supplementary Materials S1. Furthermore, goodness-of-fit plots showing predicted compared to observed plasma concentrations, AUC_last_ and C_max_ values are presented in Sections S2.2.2, S3.2.2, and S4.2.2 of Supplementary Materials S1. Overall, 82.7%, 88.9% and 89.2% of predicted plasma concentrations were within two-fold of their corresponding observed value for paroxetine, atomoxetine, and risperidone, respectively. Mean (and range) model GMFEs for paroxetine, atomoxetine and risperidone were 1.51 (1.06–3.02), 1.20 (1.00–1.88) and 1.21 (1.00–1.83) for AUC_last_ values, and 1.41 (1.01–3.64), 1.18 (1.02–1.34) and 1.21 (1.01–2.02) for C_max_ values.

### 3.3. Local Sensitivity Analysis

Local sensitivity analyses were performed using simulations after oral administrations of the respective standard doses for paroxetine (20 mg), atomoxetine (40 mg) and risperidone (2 mg). Parameters with associated sensitivity values >0.5 (100% parameter value perturbation resulting in a >50% change of predicted AUC) were considered sensitive. Here, lipophilicity (literature value) and f_u_ (literature value) were sensitive parameters for the paroxetine model. Lipophilicity (optimized value), f_u_ (literature value), CYP2D6 k_cat_ (optimized) and CYP2D6 K_M_ (literature value) were sensitive parameters for the atomoxetine model. Lipophilicity (literature value), f_u_ (literature value) and intestinal permeability (optimized value) were sensitive parameters for the risperidone model. A quantitative and visual representation of the local sensitivity analysis is provided in Sections S2.2.3, S3.2.3 and S4.2.3 of Supplementary Materials S1.

### 3.4. DGI Model Building

**Figure 4:**
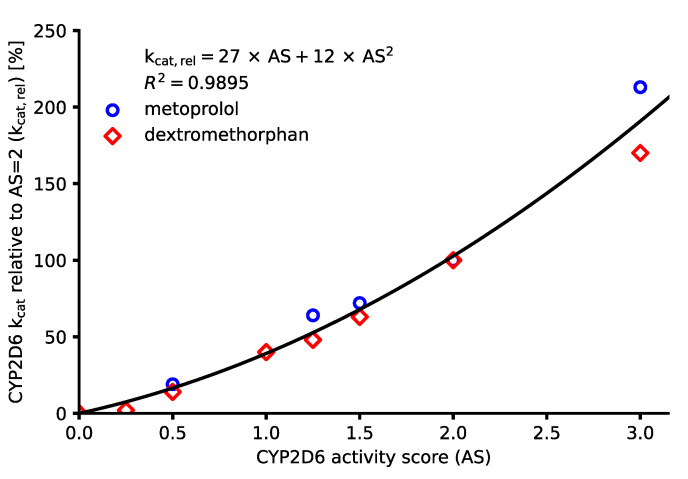
OLS regression of CYP2D6 k_cat, rel_ values for the published DGI models of metoprolol and dextromethorphan [15,17]. The solid line represents the regression curve (degree = 2, intercept = 0), and symbols represent k_cat, rel_ values for the different activity scores. AS: activity score, k_cat, rel_: k_cat_ relative to AS = 2, and R^2^: coefficient of determination.

An OLS regression of CYP2D6 k_cat, rel_ values was performed for the published metoprolol and dextromethorphan models to derive a substrate-independent scale of activity score-dependent metabolism for the newly developed models of paroxetine, atomoxetine, and risperidone. The results of the OLS regression are displayed in Figure 4.

Input values and results of the regression analysis as well as CYP2D6 model k_cat_ values for the modeled activity scores (calculated using the Equation given in Figure 4) are shown in Table 2. Here, paroxetine, atomoxetine, and risperidone CYP2D6 model k_cat_ values were calculated by multiplying interpolated k_cat, rel_ values with the optimized baseline k_cat_ value (activity score 2).

### 3.5. DGI Model Evaluation

**Figure 5:**
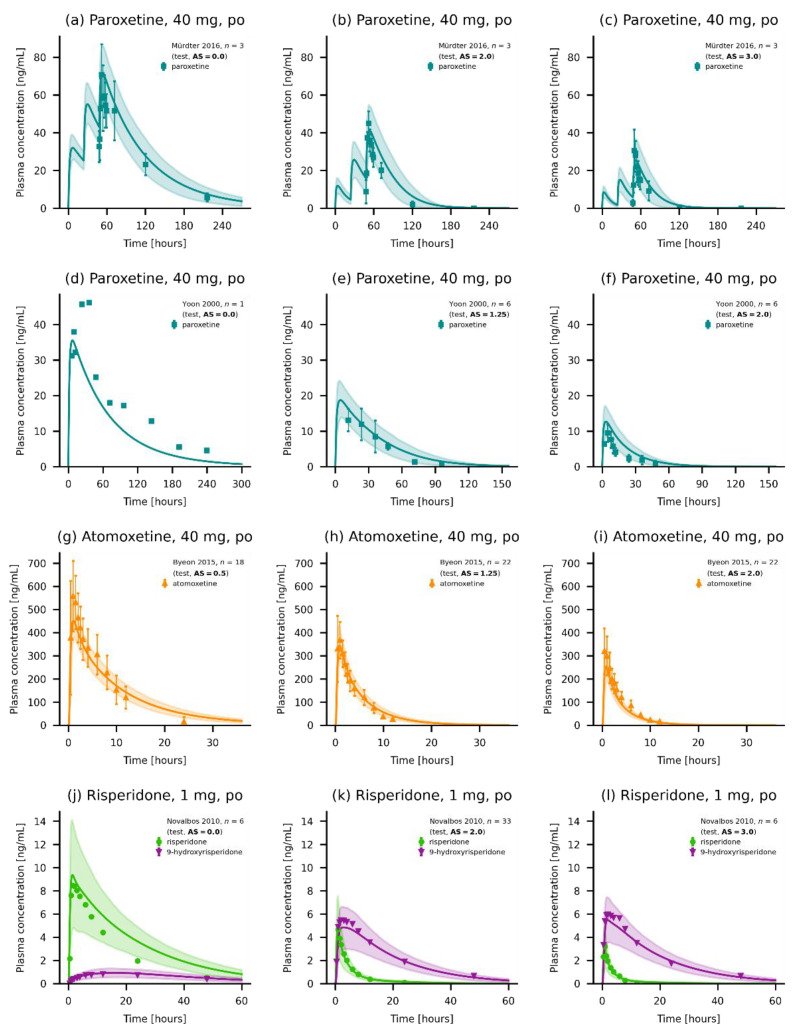
Simulated plasma concentrations of the modeled compounds for different CYP2D6 activity scores. (**a**–**f**) Paroxetine [33,40], (**g**–**i**) atomoxetine [41] and (**j**–**l**) risperidone [54] plasma concentration–time profiles of selected CYP2D6 DGI studies, compared to their observed data [33,40,41,54]. Individual predictions are shown as lines. Population predictions (*n* = 1000) are shown as lines with ribbons (arithmetic mean ± standard deviation (SD)), and symbols represent the corresponding observed data ± SD (if available).

The newly developed DGI models were evaluated using clinical studies, which stratified their subjects by CYP2D6 activity score or phenotype. These studies either provided the activity score for the investigated population, the CYP2D6 phenotype, or *CYP2D6* genotypes of all study participants. Simulations were performed using the corresponding k_cat_ values with respect to activity score (Table 2) or phenotype (Sections S2.1.1, S3.1.1 and S4.1.1 of Supplementary Materials S1). DGI model performance is presented in Figure 5, depicting representative predicted compared to observed plasma concentration–time profiles of populations with different activity scores for paroxetine (a–f), atomoxetine (g–i) and risperidone (j–l). Plots depicting the model performance of all DGI studies are presented in Sections S2.3.1, S3.3.1 and S4.3.1 of Supplementary Materials S1.

**Figure 6:**
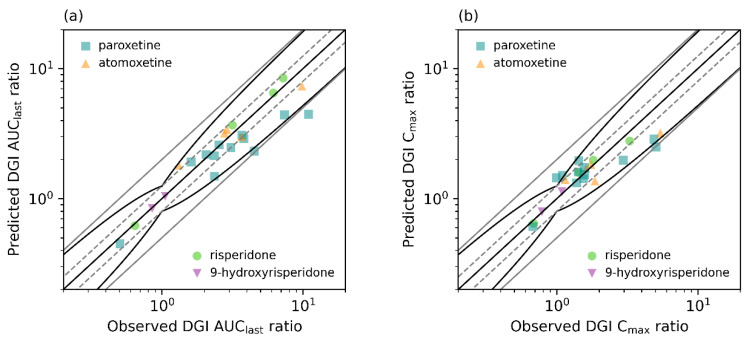
Comparison plot of predicted versus observed (**a**) DGI AUC_last_ ratios and (**b**) DGI C_max_ ratios for all analyzed CYP2D6 DGI studies. The straight black line indicates the line of identity, curved black lines show prediction success limits proposed by Guest et al., including 1.25-fold variability [58]. Solid gray lines indicate two-fold deviation, dashed gray lines show 1.25-fold deviation. AUC_last_: area under the plasma concentration–time curve from the time of the first concentration measurement to the time of the last concentration measurement, C_max_: maximum plasma concentration, and DGI: drug–gene interaction.

Overall, predicted DGI AUC_last_ and C_max_ ratios were in good agreement with observed DGI ratios, highlighting the good performance of the DGI models predicting the activity score-dependent metabolism of paroxetine, atomoxetine, and risperidone, with 22/24 DGI AUC_last_ and 22/24 C_max_ ratios within the prediction success limits proposed by Guest et al. [58] as depicted in Figure 6. The predicted DGI AUC_last_ ratios showed mean GMFEs of 1.37, 1.25 and 1.11 whereas the overall GMFEs of predicted DGI C_max_ ratios were 1.33, 1.28 and 1.16 for paroxetine, atomoxetine, and risperidone, respectively. Predicted to observed DGI AUC_last_ and C_max_ ratios for all studies are presented in Sections S2.3.3, S3.3.3, and S4.3.3 of Supplementary Materials S1.

**Figure 7:**
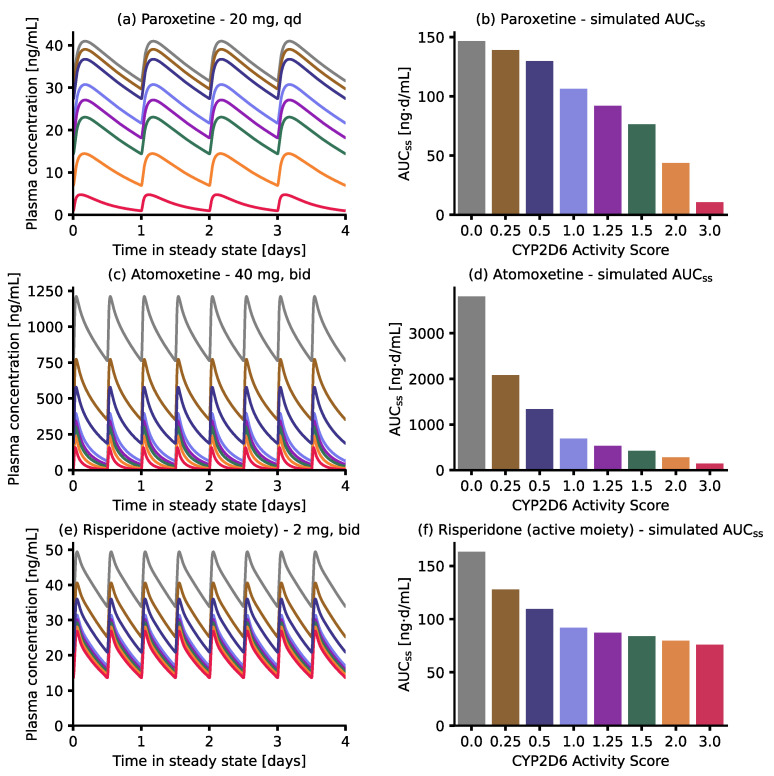
Model-based CYP2D6 DGI predictions. **Left panel**: simulations of drug exposure in individuals with different CYP2D6 activity scores after multiple oral doses of 40 mg paroxetine once daily (**a**), 40 mg atomoxetine twice daily (**c**) or 2 mg risperidone twice daily (**e**). **Right panel:** comparison of the corresponding AUC_ss_ values for the different activity scores for paroxetine (**b**), atomoxetine (**d**) and risperidone active moiety ((**f**) risperidone and 9-hydroxyrisperidone concentrations). AUC_ss_: area under the plasma concentration–time curve during steady state (calculated for days 24–28), bid: twice daily, and qd: once daily.

Simulations of steady-state plasma concentration–time profiles and AUC_ss_ values in individuals with different activity scores after multiple oral doses of 20 mg paroxetine, 40 mg atomoxetine or 2 mg risperidone and a comparison of the corresponding AUC_ss_ values are given in Figure 7. Predicted DGI AUC_ss_ ratios were 3.4, 13.6 and 2.0 for poor metabolizers (activity score 0) compared to normal metabolizers (activity score 2) for paroxetine, atomoxetine and risperidone active moiety (risperidone + 9-hydroxyrisperidone), respectively. Conversely, predicted DGI AUC_ss_ ratios were 0.2, 0.5 and 0.95 for ultrarapid metabolizers (activity score 3).

## 4. Discussion

In this study, whole-body PBPK models of paroxetine, atomoxetine, and risperidone, including its active metabolite 9-hydroxyrisperidone, are presented. A total of 57 studies were used for model building and evaluation. CYP2D6 DGIs were modeled by implementing CYP2D6 activity score-dependent metabolism of the respective compounds for activity scores ranging from 0 to 3. Parameters for the CYP2D6 activity score-dependent metabolism for the presented models were derived from previously published models [15,17] via interpolation and represent a substrate-independent approach of modeling CYP2D6 DGIs. All three models showed good performance as highlighted in the model evaluation sections.

Previously published PBPK models of paroxetine, atomoxetine and risperidone typically implemented CYP2D6 DGIs by adjusting model parameters such as CL_int_, K_M_ or k_cat_ values based on traditional CYP2D6 phenotypes (extensive and poor metabolizer) [59,60] or specific *CYP2D6* genotypes such as CYP2D6*1/*1 and CYP2D6*10/*10 [43], whereas the presented model can accurately describe both traditional phenotypes as well as CYP2D6 activity scores, allowing the models to predict compound plasma concentrations for all relevant genotypes to provide a finer graduation of CYP2D6 activity [17].

The CYP2D6 activity score-dependent metabolism was modeled by adjusting CYP2D6 k_cat_ values based on the activity score of the respective individual or population. Hence, the k_cat_ value serves as a surrogate parameter, reflecting changes in both in vivo reference concentration [61] and in vitro V_max_ [62] that typically occur due to polymorphisms in the *CYP2D6* gene. Here, K_M_ and CYP2D6 reference concentrations were fixed over the whole range of modeled activity scores and phenotypes [18]. Model CYP2D6 k_cat_ values for extensive metabolizers were consistently lower compared to normal metabolizers (activity scores 1.25–2, see Table 2 and Sections S2.1.1, S3.1.1 and S4.1.1 of Supplementary Materials S1 for paroxetine, atomoxetine and risperidone, respectively). Specifically, the CYP2D6 k_cat_ values for extensive metabolizers were 35%, 30% and 36% lower compared to activity score 1.25 for paroxetine, atomoxetine and risperidone, respectively. This is caused by the limited intersection between the aforementioned activities, mainly due to the often arbitrary definition of the extensive metabolizer phenotype [16].

While the presented approach of modeling CYP2D6 DGIs based on the activity score categories was a necessary simplification, it also represents one of the limitations of the presented study and, by extension, the activity score system itself. As suggested by van der Lee et al., CYP2D6 DGIs may be more accurately described using a continuous scale approach, reflecting the effect of *CYP2D6* allelic variants on the pharmacokinetics of CYP2D6 substrates in vivo and in vitro compared to the activity score system [9]. Additionally, certain *CYP2D6* alleles have been described to display substrate-specific effects in vitro and in vivo. For instance, the CYP2D6*17 allele, classified as a reduced function (activity score 0.5) allele, shows increased activity in risperidone metabolism when compared to the wildtype *1 allele [63]. These effects are not considered in the classification of alleles using the activity score system [61]. Regardless, current clinical guidelines by the Dutch Pharmacogenetics Working Group (DPWG), the Clinical Pharmacogenetics Implementation Consortium (CPIC) and other major institutions in this field, are based on the activity score system [5]. Moreover, a allele-specific modeling approach would drastically increase model complexity and would require an extensive amount of in vitro and in vitro model input data [18]. Hence, the approach presented in this study was deemed a more practical choice in the context of PBPK modeling.

The presented paroxetine model includes metabolism via CYP2D6 and CYP3A4 and an unspecific hepatic clearance pathway as a surrogate pathway for metabolism via other CYP enzymes that were reported to metabolize paroxetine in vitro [22]. Here, additional experimental in vitro data such as K_M_ and V_max_ values were available for paroxetine metabolism via CYP1A2, CYP2C19 and CYP3A5. However, these enzymes were described to have a smaller effect on paroxetine kinetics compared to CYP2D6 and CYP3A4 [22]. Furthermore, CYP3A4 was implemented to describe the effect of auto-inhibition via CYP3A4 on the pharmacokinetics of paroxetine [23,55], especially in poor metabolizers of CYP2D6. No metabolite of paroxetine was implemented in the model due to a lack of reported metabolite plasma concentrations in the published literature, presumably due to the chemical and metabolic instability of major metabolite paroxetine-catechol [22,35]. Furthermore, paroxetine has been suggested as a substrate of P-glycoprotein (P-gp) in the published literature [39,64]. However, while a moderate affinity of paroxetine to P-gp was observed in in vitro experiments [65], genetic polymorphisms in the *ABCB1* gene were described to have no significant on paroxetine plasma concentrations in vivo [66]. Hence, the authors did not implement active transport via P-gp in the model. Regardless, the model was able to describe paroxetine plasma concentrations for all doses (20–70 mg) in published clinical studies.

CYP2D6 has been described to be mainly responsible for atomoxetine metabolism, as atomoxetine AUC was found to be increased by 400% in poor metabolizers of CYP2D6 compared to extensive metabolizers [45]. In the presented PBPK model, atomoxetine metabolism was described by implementing CYP2D6 and CYP2C19. Although 4-hydroxyatomoxetine has been reported to be primarily formed via CYP2D6, only a total of four plasma concentration–time profiles of 4-hydroxyatomoxetine were reported in the published literature [41,67]. Thus, the metabolite was not explicitly modeled. However, as more clinical studies reporting 4-hydroxyatomoxetine plasma concentrations become available, the presented PBPK model of atomoxetine can be extended to cover the formation of 4-hydroxyatomoxetine.

While CYP1A2, CYP2B6, CYP2C9 and CYP3A4 were also described to contribute to the metabolism of atomoxetine in vitro, their relative contribution to atomoxetine depletion was found to be far smaller compared to CYP2C19 [24]. Hence, CYP2C19 serves as a surrogate pathway for multiple CYP enzymes in the presented model. While CYP2C19 is also polymorphically expressed, and CYP2C19 DGIs in CYP2C19 have been described in the literature [68], they were considered negligible, as CYP2D6 accounts for approximately 90% of atomoxetine oral clearance in normal metabolizers of CYP2D6 [45] and the CYP2C19 k_cat_ value was below the sensitivity threshold for atomoxetine model parameters (see Section S3.2.3 of Supplementary Materials S1).

To describe the activity score-dependent metabolism of risperidone, an established parent-metabolite model was used [21] and adapted. The model includes metabolism via CYP2D6 and CYP3A4 for risperidone as well as active transport via P-gp for both risperidone and its active metabolite 9-hydroxyrisperidone [69].

Simulations of steady-state plasma concentrations for the modeled compounds revealed that, although k_cat, rel_ values implemented in the respective DGI models for the different activity scores were the same, AUC_ss_ values behaved differently between the three compounds. For instance, AUC_ss_ DGI ratios for CYP2D6 poor metabolizers (activity score 0) were 3.4, 13.6 and 2.0, whereas AUC_ss_ DGI ratios for ultrarapid metabolizers (activity score 3) were 0.2, 0.5 and 0.95 for paroxetine, atomoxetine and risperidone, respectively. Here, different model-specific factors might influence predicted AUC_ss_ DGI ratios. For risperidone, the total active moiety was considered (risperidone + 9-hydroxyrisperidone). Here, a decrease in the risperidone AUC with increasing activity scores typically infers an increase in 9-hydroxyrisperidone AUC, partially compensating the effect of CYP2D6 DGIs on the AUC of the total active moiety, and the overall pharmacodynamic effect of risperidone, as 9-hydroxyrisperidone has similar activity compared to risperidone [70]. Conversely, the paroxetine model includes auto-inhibition via mechanism-based inhibition of CYP2D6 and CYP3A4, reducing the differences between DGI AUC_ss_ values for the different modeled activity scores.

## 5. Conclusions

This study presents whole-body PBPK models of paroxetine, atomoxetine, and risperidone. The models implement CYP2D6 activity score-dependent metabolism informed from previously published PBPK models of CYP2D6 substrates and have been successfully used to predict the plasma concentrations of their model compounds both in non-DGI and DGI scenarios with various CYP2D6 activity scores. The final PBPK model files will be made publicly available in the Clinical Pharmacy Saarland University GitHub model repository (http://models.clinicalpharmacy.me/). Due to the mechanistic implementation of human physiology and important pharmacokinetic pathways, the models allow for knowledge-based scaling to special populations and can serve as the basis for future investigations of CYP2D6 drug-drug–gene interaction (DDGI) scenarios.

## Figures and Tables

**Table 2:** OLS regression input values and interpolated k_cat, rel_ values alongside model CYP2D6 k_cat_ values for paroxetine, atomoxetine, and risperidone for different CYP2D6 activity scores.

CYP2D6 Activity Score	CYP2D6 k_cat, rel_ [%]			CYP2D6 k_cat_ [1/min] ^a^			
	MET	DEX	INTRPL	PAR	ATO	RIS_9HR_	RIS_other_
0	0	0	0	0.00	0.00	0	0.00
0.25	-	2	8	0.30	7.63	0.23	0.14
0.5	19	14	17	0.66	16.79	0.52	0.31
0.75	-	-	27	1.08	27.48	0.84	0.51
1	-	40	39	1.56	39.70	1.22	0.74
1.25	64	48	53	2.11	53.44	1.64	1.00
1.5	72	63	68	2.71	68.70	2.11	1.29
2	100	100	102	4.09 ^b^	103.82 ^b^	3.19 ^b^	1.94 ^b^
3	213	170	189	7.58	192.37	5.91	3.60

-: not available, ^a^: values calculated as the product of the relative k_cat_ value and the optimized k_cat_ for populations with an activity score of 2, ^b^: optimized value, ATO: atomoxetine, DEX: dextromethorphan, INTRPL: interpolated values using the polynomial equation of the OLS regression, k_cat, rel_: k_cat_ relative to AS = 2, MET: metoprolol, PAR: paroxetine, RIS_9HR_: risperidone → 9-hydroxyrisperidone, and RIS_other_: risperidone → other metabolites.

## Data Availability

All modeling files, including the clinical study data utilized, can be found at http://models.clinicalpharmacy.me/.

## Supplementary Materials

The following supporting information can be downloaded at: www.mdpi.com/article/10.3390/pharmaceutics14081734/s1, Supplementary Materials S1: Methods (Addendum); Supplementary Materials S2: Paroxetine; Supplementary Materials S3: Atomoxetine; Supplementary Materials S4: Risperidone; Supplementary Materials S5: Abbreviations. References [71,72,73,74,75,76,77,78,79,80,81,82,83,84,85,86,87,88,89,90,91,92,93] are cited in the Supplementary Materials.
