# Supplementary material for: Physiologically Based Pharmacokinetic Modeling to Describe the CYP2D6 Activity Score-Dependent Metabolism of Paroxetine, Atomoxetine and Risperidone
Source: Pharmaceutics. 2022 Aug 18;14(8):1734. doi: 10.3390/pharmaceutics14081734 (PMC9414337; doi:10.3390/pharmaceutics14081734)
Supplement: Supplementary file 1 [file pharmaceutics-14-01734-s001.zip › pharmaceutics-1836000-supplementary.pdf]

pharmaceutics

Supplementary Materials

Physiologically Based Pharmacokinetic Modeling to Describe the CYP2D6 Activity Score-Dependent Metabolism of Paroxetine, Atomoxetine and Risperidone

Simeon Rüdesheim[1,2], Dominik Selzer[1], Thomas Mürdter[2], Svetlana Igel[2], Reinhold Kerb[2], Matthias Schwab[2,3,4] and Thorsten Lehr[1,*]

1 Department of Clinical Pharmacy, Saarland University, 66123 Saarbrücken, Germany

2 Dr. Margarete Fischer-Bosch-Institute of Clinical Pharmacology, 70376 Stuttgart, Germany

3 Departments of Clinical Pharmacology, Pharmacy and Biochemistry, University Tübingen, 72076 Tübingen, Germany

4 Cluster of Excellence iFIT (EXC2180) "Image-Guided and Functionally Instructed Tumor Therapies", University of Tübingen, 72076 Tübingen, Germany

* Correspondence: thorsten.lehr@mx.uni-saarland.de; Tel.: +49-681-302-70255

Received: 11 July 2022; Accepted: 17 August; Published: 18 August 2022

Corresponding Author Prof. Dr. Thorsten Lehr
Clinical Pharmacy, Saarland University
Campus C5 3, 66123 Saarbrücken
Phone: +49 681 302 70255
Email: thorsten.lehr@mx.uni-saarland.de
ORCID: 0000 0002 8372 1465

Contents

S1 Methods (Addendum) 3
S1.1 Virtual Individuals . . . 3
S1.2 Virtual Populations . . . 3
S1.3 System-Dependent Parameters . . . 4
S1.4 PBPK Model Sensitivity Analysis . . . 4

S2 Paroxetine 5
S2.1 Paroxetine PBPK Base Model Building . . . 5
S2.1.1 Paroxetine Drug-Dependent Parameters . . . 5
S2.1.2 Paroxetine Clinical Studies . . . 6
S2.2 Paroxetine PBPK Base Model Evaluation . . . 7
S2.2.1 Plasma Concentration-Time Profiles . . . 7
S2.2.2 Goodness-of-Fit Plots . . . 9
S2.2.3 Sensitivity Analysis . . . 10
S2.3 Paroxetine DGI Model Evaluation . . . 11
S2.3.1 Plasma Concentration-Time Profiles . . . 11
S2.3.2 Goodness-of-Fit Plots . . . 14
S2.3.3 DGI Ratios . . . 15

S3 Atomoxetine 16
S3.1 Atomoxetine PBPK Base Model Building . . . 16
S3.1.1 Drug-dependent Parameters . . . 16
S3.1.2 Clinical studies . . . 17
S3.2 Atomoxetine PBPK Base Model Evaluation . . . 18
S3.2.1 Plasma Concentration-Time Profiles . . . 18
S3.2.2 Goodness-of-Fit Plots . . . 19
S3.2.3 Sensitivity Analysis . . . 20
S3.3 Atomoxetine DGI Model Evaluation . . . 20
S3.3.1 Plasma Concentration-Time Profiles . . . 20
S3.3.2 Goodness-of-Fit Plots . . . 23
S3.3.3 DGI ratios . . . 24

S4 Risperidone 25
S4.1 Risperidone PBPK Base Model Building . . . 25
S4.1.1 Drug-dependent parameters . . . 25
S4.1.2 Clinical studies . . . 27
S4.2 Risperidone PBPK Base Model Evaluation . . . 28
S4.2.1 Plasma Concentration-Time Profiles . . . 28
S4.2.2 Goodness-of-Fit Plots . . . 29
S4.2.3 Sensitivity Analysis . . . 31
S4.3 Risperidone DGI Model Evaluation . . . 32
S4.3.1 Plasma Concentration-Time Profiles . . . 32
S4.3.2 Goodness-of-Fit Plots . . . 34
S4.3.3 DGI Ratios . . . 36

S5 Abbreviations 37

S1 Methods (Addendum)

S1.1 Virtual Individuals

The PBPK model was built based on data from healthy individuals, using the reported sex, ethnicity and mean values for age, weight and height from each study protocol. If no demographic information was provided, the following default values were substituted: male, European, 30 years of age, 73 kg body weight and 176 cm body height (characteristics from the PK-Sim® population database [34, 48, 50]. CYP2D6 was implemented in accordance with literature, using the PK-Sim® expression database to define their relative expression in the different organs of the body [37]. Details on the implementation of CYP2D6 are summarized in Section S1.3.

S1.2 Virtual Populations

For population simulations, virtual populations of 1000 individuals were created based on the population characteristics stated in the respective publication. If no information was provided in the publication, populations based on European male individuals aged 20–50 years were assumed. Metrics were generated (depending on ethnicity) from one of the following databases; American: NHANES [34] database, Asian: Tanaka model [48], European: ICRP database [50]. In the generated virtual populations, system-dependent parameters such as weight, height, organ volumes, blood flow rates, tissue compositions, etc. were varied by the implemented algorithm in PK-Sim® within the limits of the databases listed above [34, 48, 50]. Since study populations were grouped by their AS or phenotype, no variability in CYP2D6 reference concentrations was assumed for population simulations. Reference concentrations of implemented proteins as well as the relative expression are provided in Section S1.3.

S1.3 System-Dependent Parameters

Table S1.3.1: System-dependent parameters

	Reference concentration			Localization	Half-life	
	Mean†	GSD*	Relative expression[a]		Liver [h]	Intestine [h]
Enzymes						
CYP2C19	0.76 [41]	1.79 [37]	RT-PCR [37]	Intracellular	26 [37]	23 [37]
CYP2D6	0.40 [41]	0[b]	RT-PCR [37]	Intracellular	51 [37]	23 [37]
CYP3A4	4.32 [41]	1.18 [37]	RT-PCR [37]	Intracellular	36 [42]	23 [13]
Transporters						
P-gp	1.41[15]	1.60 [38]	RT-PCR [35]	Apical (Efflux)	36 [37]	23 [37]

†: µmol protein/l in the tissue of highest expression, *: Geometric standard deviation of the reference concentration, [a]: In the different organs (PK-Sim expression database profile), [b]: Variability for Cytochrome P450 2D6 (CYP2D6) was set to 0, as study populations were stratified by CYP2D6 activity,

S1.4 PBPK Model Sensitivity Analysis

Sensitivity of the final models to single parameter changes (local sensitivity analysis) was calculated as relative change of the $\mathrm{AUC}_{0\text{–}24\ \mathrm{h}}$. Sensitivity analysis was carried out using a relative perturbation of 1000% (variation range 10.0, maximum number of 9 steps). Parameters were included into the analysis if they have been optimized, if they are associated with optimized parameters or if they might have a strong impact due to calculation methods used in the model. Sensitivity to a parameter was calculated as the ratio of the relative change of the simulated area under the plasma concentration-time curve (AUC) from the time of the drug administration extrapolated to infinity ($\mathrm{AUC}_{0\text{–inf}}$) to the relative variation of the parameter according to Eq. S1:

$$S = \frac{\Delta AUC_{0--inf}}{\Delta p} \times \frac{p}{AUC_{0--inf}} \tag{S1}$$

where S = sensitivity of the $AUC_{0-24\ h}$ to the examined model parameter, ΔAUC_{0--inf} = change of the $\mathrm{AUC}_{0\text{–inf}}$, $AUC_{0-24\ h}$ = simulated $\mathrm{AUC}_{0\text{–inf}}$ with the original parameter value, Δp = change of the examined parameter value, p = original parameter value.

A sensitivity of +0.5 signifies that a 100% increase of the examined parameter value causes a 50% increase of the simulated $\mathrm{AUC}_{0\text{–}24\ \mathrm{h}}$.

S2 Paroxetine

S2.1 Paroxetine PBPK Base Model Building

S2.1.1 Paroxetine Drug-Dependent Parameters

Table S2.1.1: Drug-dependent parameters for the final paroxetine PBPK model

Parameter	Unit	Value	Source	Literature	Reference
MW	g/mol	329.37	Literature	329.37	[57]
pKa (base)	-	9.90	Literature	9.90	[1]
Solubility (pH 4.5)	mg/mL	7.31	Literature	7.31	[20]
logP	-	3.95	Literature	3.95	[1]
f_u	%	5.00	Literature	5.00	[19]
CYP3A4 K_M	µmol/L	4.70	Literature	$4.70^{\dagger}$	[17]
CYP3A4 k_{cat}	1/min	1.01	Optimized	5.32	[17]
CYP2D6 K_M	µmol/L	0.03	Literature	$0.03^{\dagger}$	[17]
CYP2D6 k_{cat} ^{EM}	1/min	1.37	Optimized	9.70	[17]
CYP2D6 k_{cat} ^{PM}	1/min	0.00	Assumed	-	[17]
Unspecific CL_{hep}	1/min	1.37	Optimized	-	[17]
CYP2D6 K_i	µmol/L	0.17	Optimized	0.32	[52]
CYP2D6 k_{inact}	1/min	0.17	Literature	0.17	[52]
CYP3A4 K_i	µmol/L	4.48	Literature	$4.48^{\dagger}$	[5]
CYP3A4 k_{inact}	1/min	0.01	Literature	0.01	[5]
GFR fraction	-	1.00	Assumed	-	-
CR Weibull shape	-	7.17	Optimized	-	-
CR Weibull time	min	276.35	Optimized	-	[9, 21]
Partition coefficients	-	Diverse	Calculated	R&R	[40]
Cellular permeabilities	cm/min	0.28	Calculated	PK-Sim	[18]
Specific intestinal perm.	cm/min	3.93E-05	Calculated	4.89E-04	[18]

-: not given, †: in vitro values corrected for binding in the assay fu_{mic} calculated according to [2].

S2.1.2 Paroxetine Clinical Studies

Table S2.1.2: Paroxetine study table

Route	Dose [mg]	n	Females [%]	Age [years]	Weight [kg]	CYP2D6 activity	Dataset	References
PBPK base model building and evaluation								
iv (inf, sd)	28	1	0	28	75	-	training	Lund 1982 [25]
iv (inf, sd)	28	1	0	24	66	-	training	Lund 1982 [25]
iv (inf, sd)	28	1	0	26	88	-	training	Lund 1982 [25]
iv (inf, sd)	23	1	0	29	72	-	training	Lund 1982 [25]
po (tab, qd)	20	22	23	38 (20-49)	-	g-EM	training	Belle 2002 [3]
po (-, qd)	20	25	64	26	64	-	test	Calvo 2004 [8]
po (po, sd)	45	1	0	28	75	-	training	Lund 1982 [25]
po (po, sd)	45	1	0	24	66	-	training	Lund 1982 [25]
po (po, sd)	45	1	0	26	88	-	training	Lund 1982 [25]
po (po, sd)	45	1	0	29	72	-	training	Lund 1982 [25]
po (tab, sd)	20	28	0	28 (18-42)	72 (57-87)	-	training	Massaroti 2005 [29]
po (-, sd)	70	5	0	31 (22-44)	-	-	test	McClelland 1984 [30]
po (-, qd)	20	14	14	34 (19-55)	75	-	test	Schoedel 2012 [44]
po (tab, qd)	20	7	0	23	65	p-EM	test	Segura 2005 [45]
po (tab, qd)	20	26	69	44 (18-64)	69 (51-89)	g-EM	test	van der Lee 2007 [51]
po (-, sd)	20	12	25	25 (20-35)	58 (46-75)	AS = 1.25*	test	Yasui-Furukori 2006 [54]
po (-, sd)	20	13	23	24 (21-35)	57 (45-67)	-	test	Yasui-Furukori 2007 [53]
DGI model building and evaluation								
po (CR, sd)	25	4	25	26 (19–45)	64	AS = 0.5*	test	Chen 2015 [9]
po (CR, sd)	25	11	45	26 (19–45)	61	AS = 1.0*	test	Chen 2015 [9]
po (CR, sd)	25	5	60	22 (19–45)	58	AS = 1.5*	test	Chen 2015 [9]
po (CR, sd)	25	4	25	28 (19–45)	61	AS = 2*	test	Chen 2015 [9]
po (tab, sd)	40	3	100	25 (22–26)	62 (50–70)	AS = 0*	test	Mürdter 2016 [12, 16, 31]
po (tab, sd)	40	4	100	24 (21–20)	59 (56–64)	AS = 0.5*	test	Mürdter 2016 [12, 16, 31]
po (tab, sd)	40	1	100	25	68	AS = 0.75*	test	Mürdter 2016 [12, 16, 31]
po (tab, sd)	40	2	100	26 (23–28)	67 (64–74)	AS = 1*	test	Mürdter 2016 [12, 16, 31]
po (tab, sd)	40	3	100	32 (26–43)	57 (48–64)	AS = 2*	training	Mürdter 2016 [12, 16, 31]
po (tab, sd)	40	3	100	26 (22–28)	62 (54–73)	AS = 3*	test	Mürdter 2016 [12, 16, 31]
po (tab, qd)	30	8	0	27 (23–39)	82 (68–95)	p-PM	training	Sindrup 1992 [46]
po (tab, qd)	30	9	0	24 (20–30)	73 (65–81)	p-EM	training	Sindrup 1992 [46]
po (tab, sd)	40	1	100	21	58	AS = 0*	test	Yoon 2000 [55]
po (tab, sd)	40	3	0	22	68	AS = 0.5*	test	Yoon 2000 [55]
po (tab, sd)	40	6	0	22	67	AS = 1.25*	test	Yoon 2000 [55]
po (tab, sd)	40	6	17	23	59	AS = 2*	training	Yoon 2000 [55]

-: not given, *: full genotype provided in publication.

S2.2 Paroxetine PBPK Base Model Evaluation

S2.2.1 Plasma Concentration-Time Profiles

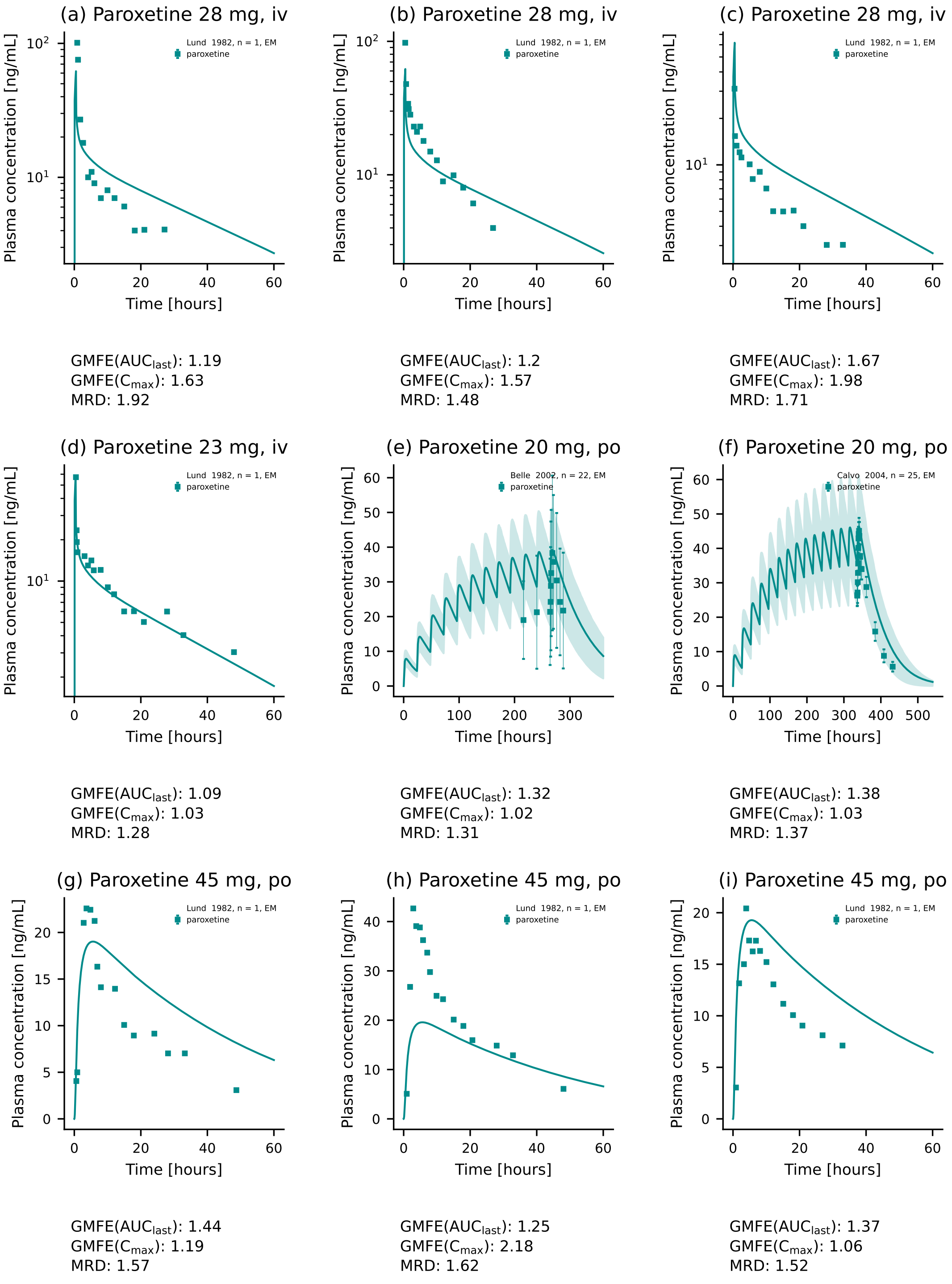

Figure S2.2.1: Paroxetine plasma concentration-time profiles. Population predictions (n=1000) are shown as lines with ribbons (arithmetic mean ± SD). Individual predictions (n=1) are shown as lines. Symbols represent the corresponding observed data ± SD if provided.

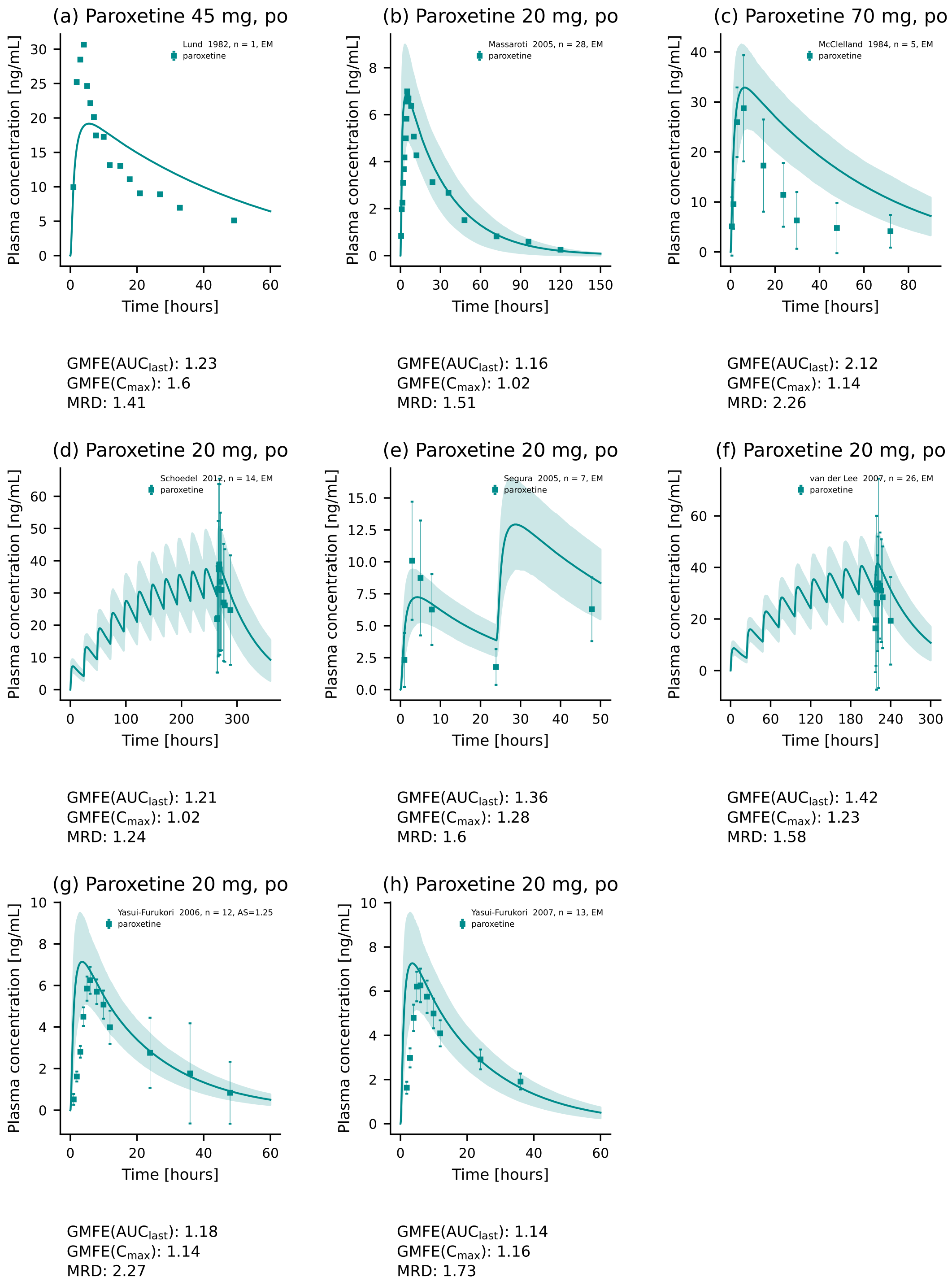

Figure S2.2.2: Paroxetine plasma concentration-time profiles. Population predictions (n=1000) are shown as lines with ribbons (arithmetic mean ± SD). Individual predictions (n=1) are shown as lines. Symbols represent the corresponding observed data ± SD if provided.

S2.2.2 Goodness-of-Fit Plots

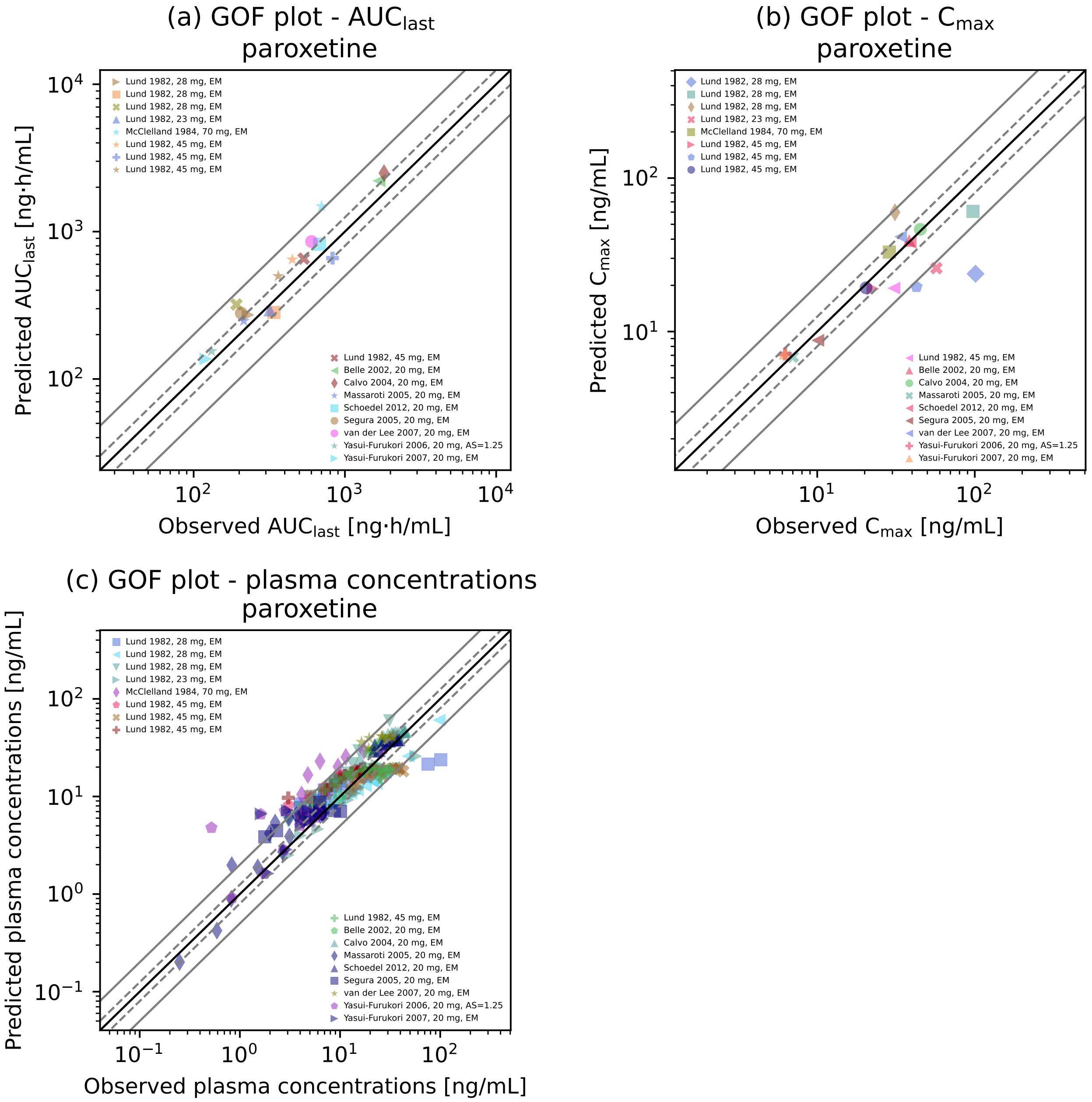

Figure S2.2.3: Goodness of fit plots. Predicted versus observed (a) AUC_{last}, (b) C_{max} and (c) plasma concentration values for all studies. The solid black line marks the line of identity, the dashed grey lines mark the 0.8- to 1.25-fold range, the solid grey lines indicate the 0.5- to 2-fold range. Colored symbols represent the study population given in the legend.

S2.2.3 Sensitivity Analysis

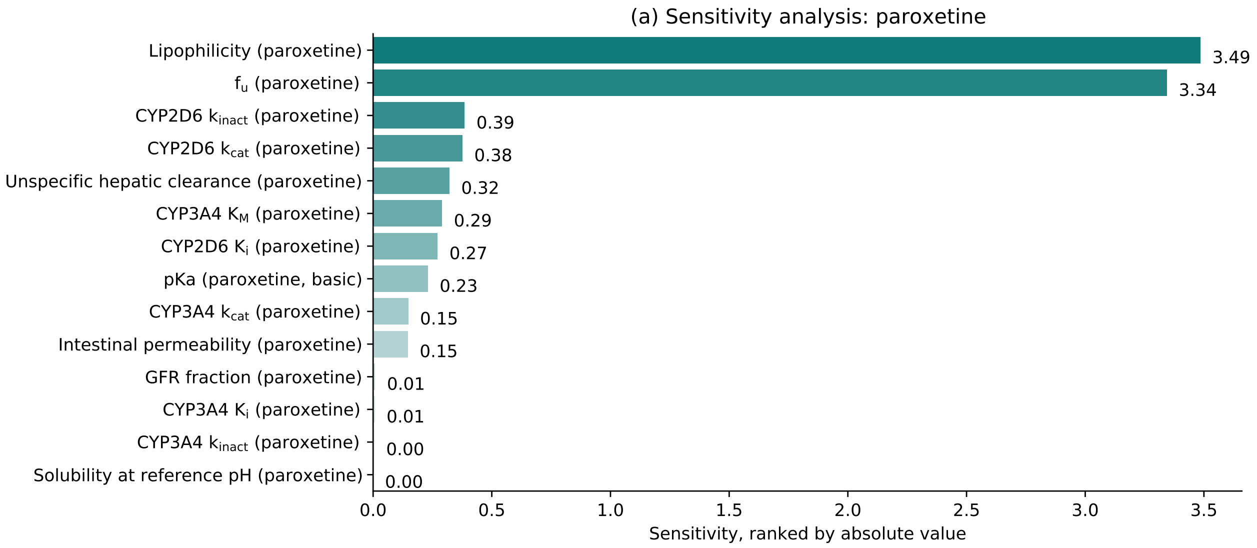

Figure S2.2.4: Sensitivity analysis of the paroxetine model. Sensitivity of the model to single parameters, determined as change of the simulated AUC from time of the administration extrapolated to infinity of a single oral administration of 20 mg paroxetine hydrochloride.

S2.3 Paroxetine DGI Model Evaluation

S2.3.1 Plasma Concentration-Time Profiles

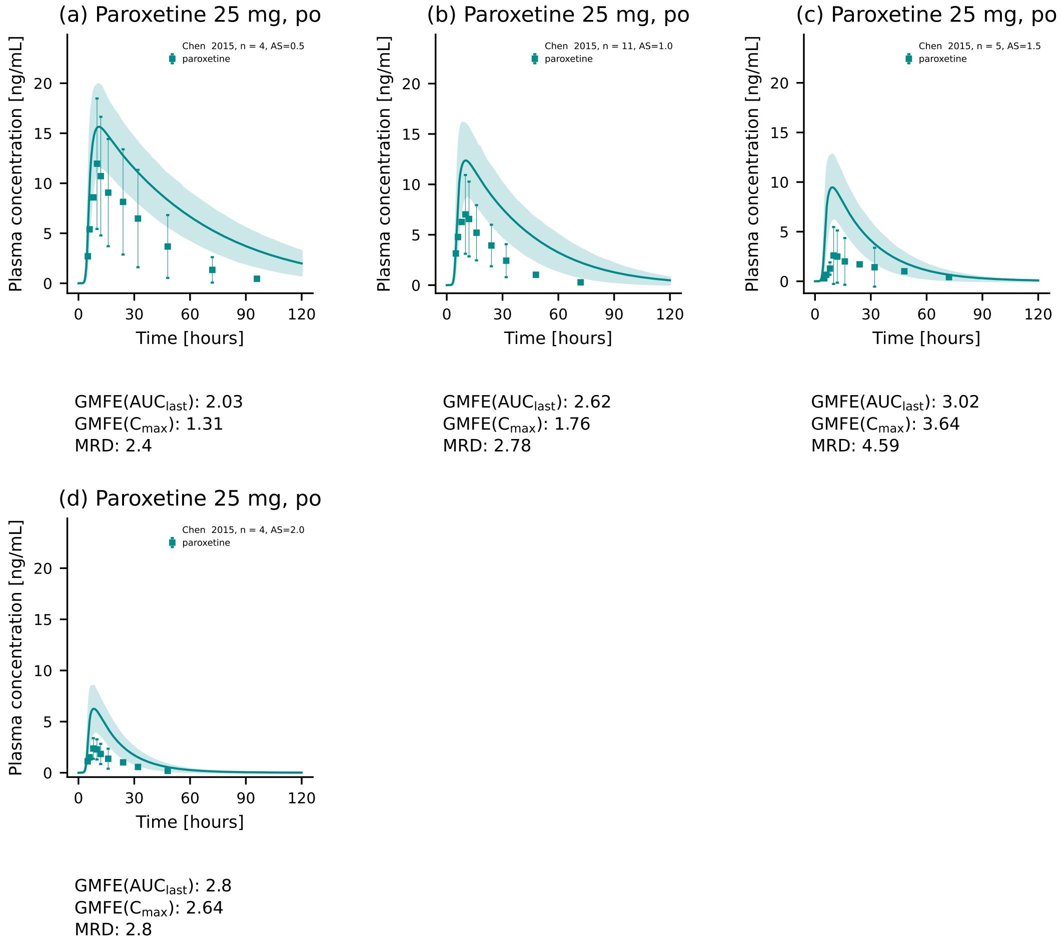

Figure S2.3.5: Paroxetine plasma concentration-time profiles [9]. Population predictions (n=1000) are shown as lines with ribbons (arithmetic mean ± SD). Symbols represent the corresponding observed data ± SD if provided.

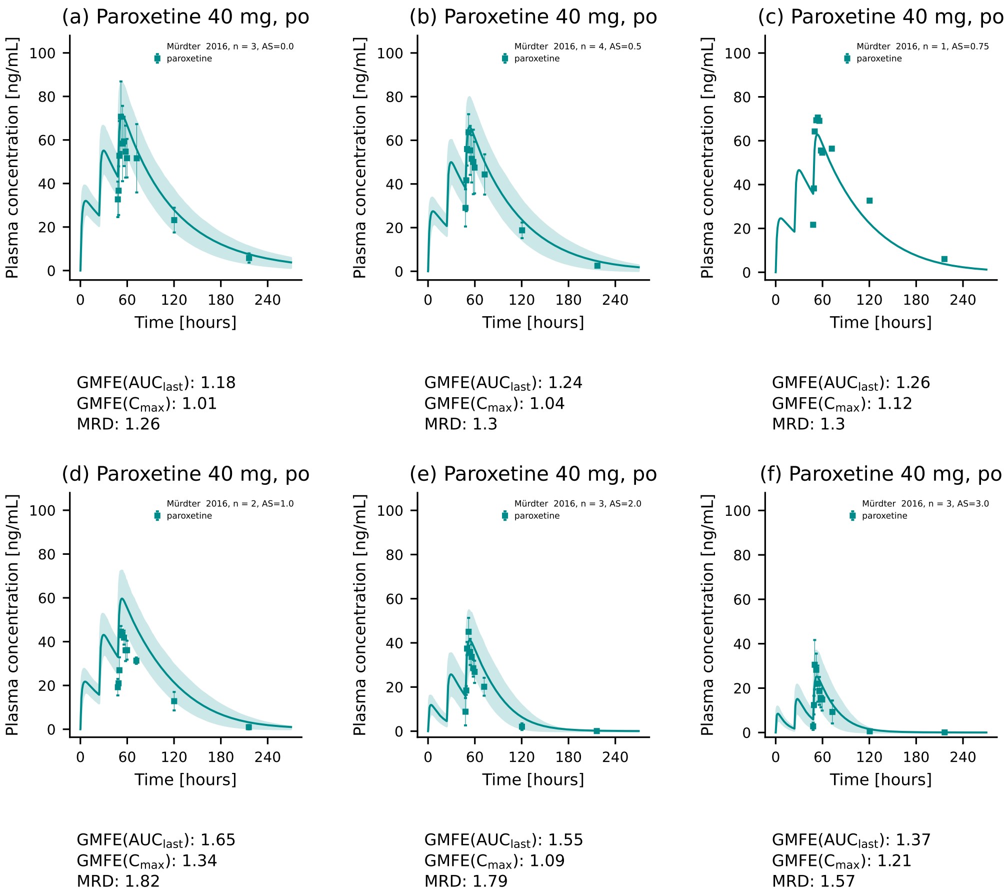

Figure S2.3.6: Paroxetine plasma concentration-time profiles [12]. Population predictions (n=1000) are shown as lines with ribbons (arithmetic mean ± SD). Individual predictions (n=1) are shown as lines. Symbols represent the corresponding observed data ± SD if provided.

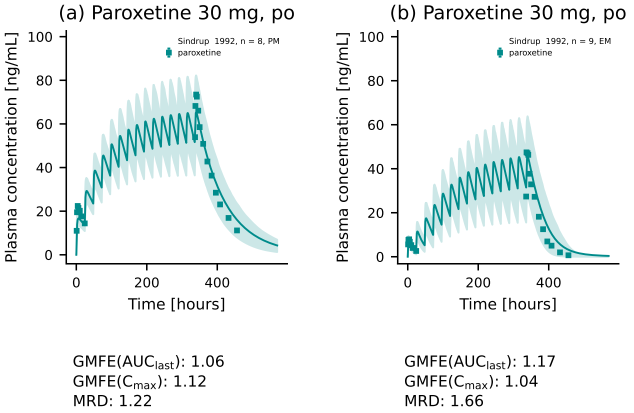

Figure S2.3.7: Paroxetine plasma concentration-time profiles [46]. Population predictions (n=1000) are shown as lines with ribbons (arithmetic mean ± SD). Symbols represent the corresponding observed data ± SD if provided.

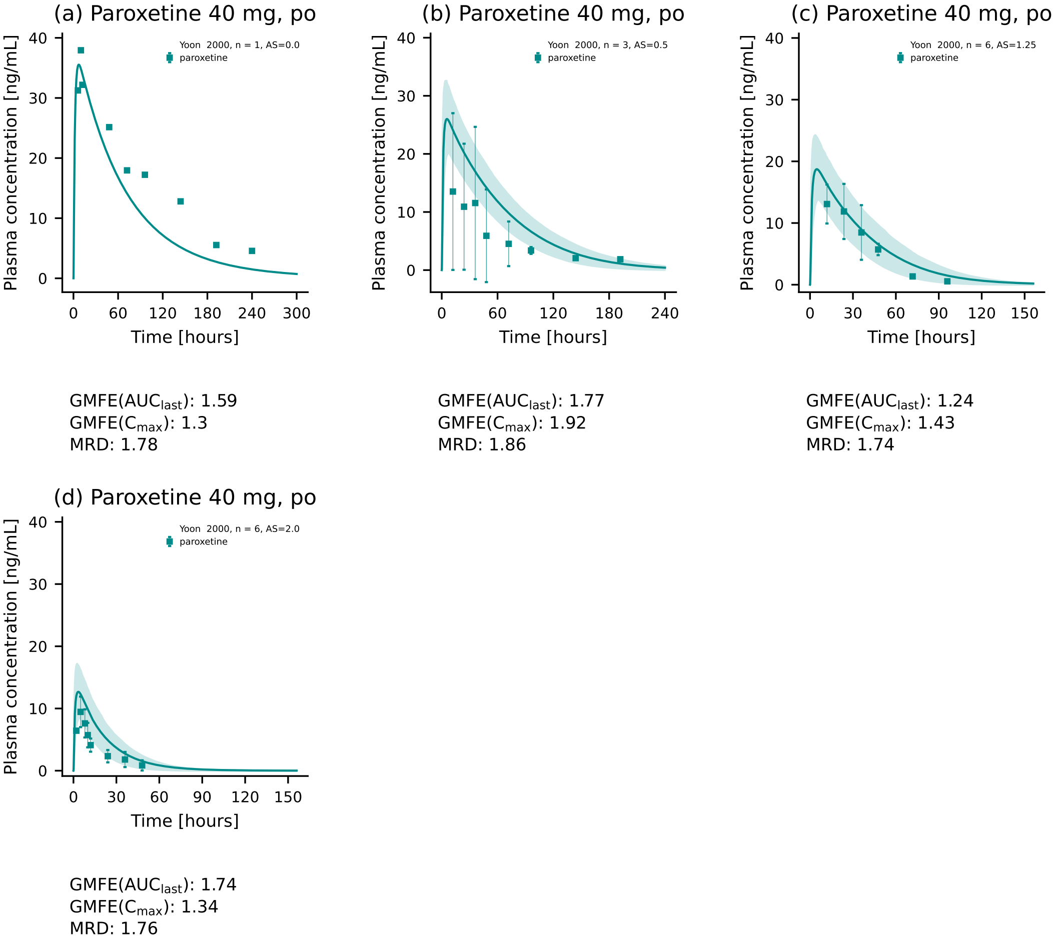

Figure S2.3.8: Paroxetine plasma concentration-time profiles [55]. Population predictions (n=1000) are shown as lines with ribbons (arithmetic mean ± SD). Individual predictions (n=1) are shown as lines. Symbols represent the corresponding observed data ± SD if provided.

S2.3.2 Goodness-of-Fit Plots

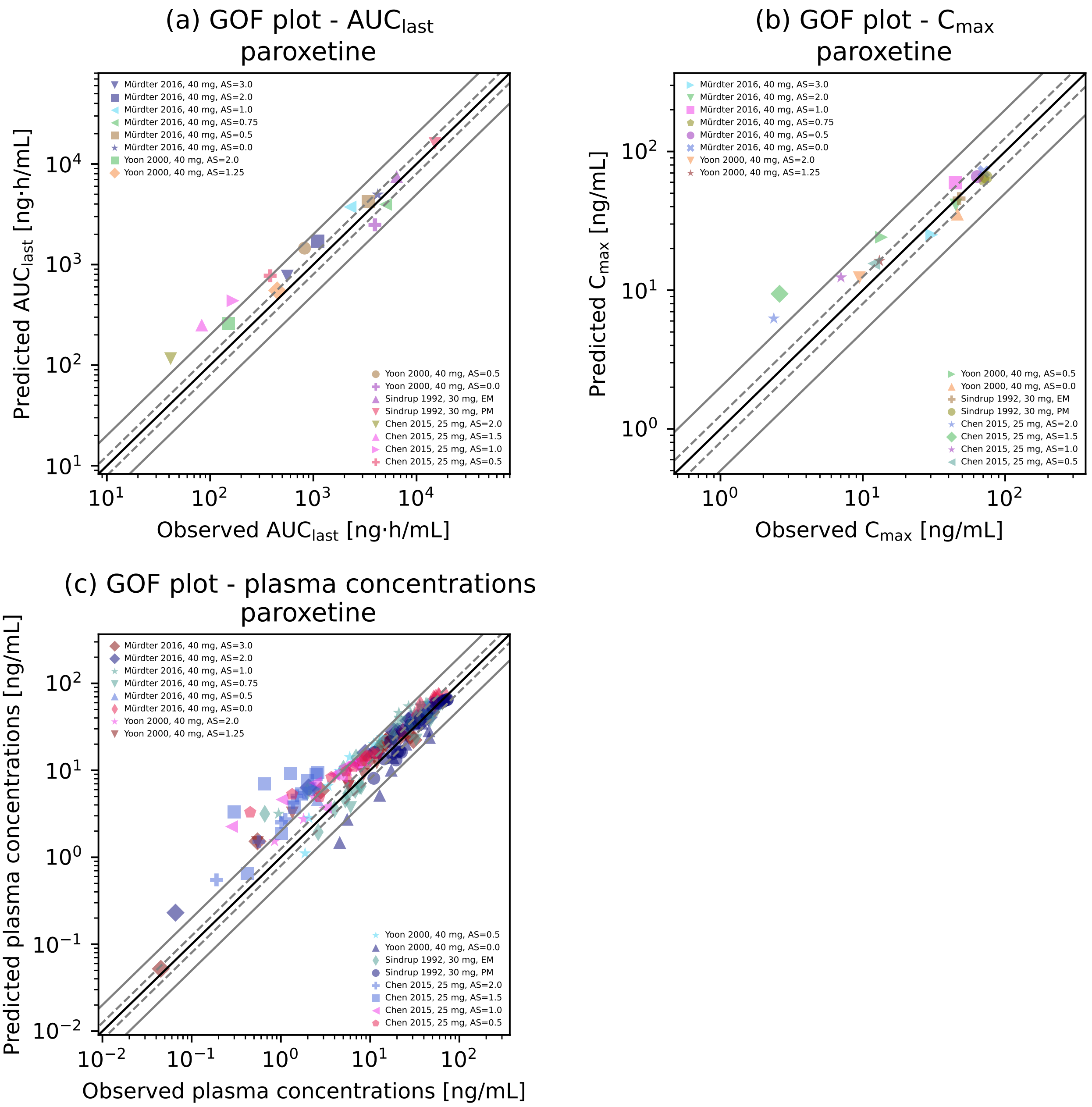

Figure S2.3.9: Goodness of fit plots. Predicted versus observed (a) AUC_{last}, (b) C_{max} and (c) plasma concentration values for all DGI studies. The solid black line marks the line of identity, the dashed grey lines mark the 0.8- to 1.25-fold range, the solid grey lines indicate the 0.5- to 2-fold range. Colored symbols represent the study population given in the legend.

S2.3.3 DGI Ratios

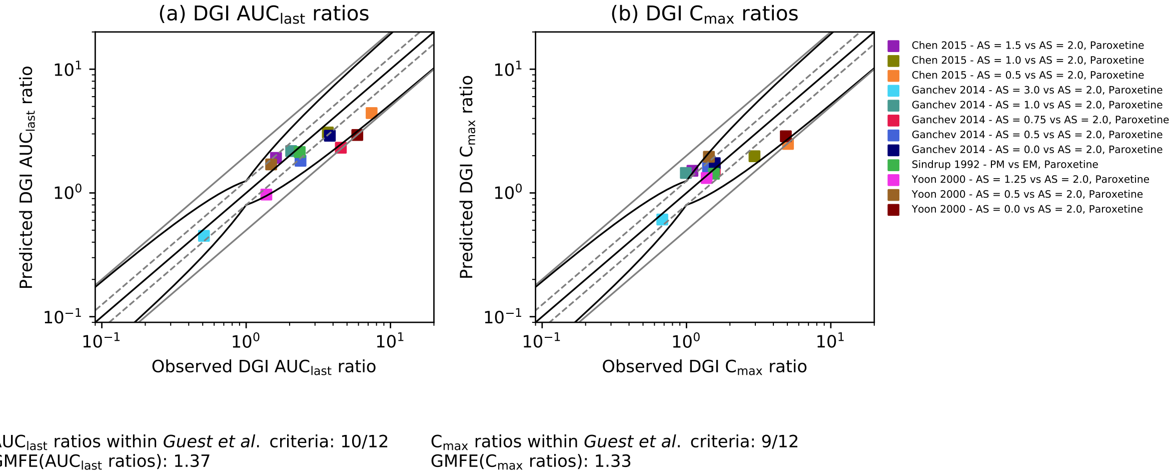

Figure S2.3.10: DGI ratio plot. Predicted versus observed (a) DGI AUC_{last} and (b) C_{max} ratios for all DGI studies. The solid straight black line marks the line of identity, the solid curved black line shows the prediction success limits proposed by *Guest et al.* [14], the dashed grey lines mark the 0.8- to 1.25-fold range, the solid grey lines indicate the 0.5- to 2-fold range. Colored symbols represent the study population given in the legend.

S3 Atomoxetine

S3.1 Atomoxetine PBPK Base Model Building

S3.1.1 Drug-dependent Parameters

Table S3.1.1: Drug-dependent parameters for the final atomoxetine PBPK model

Parameter	Unit	Value	Source	Literature	Reference
MW	g/mol	255.35	Literature	255.35	[57]
pKa (base)	-	9.80	Literature	9.80	[47]
Solubility (pH 7.4)	mg/mL	10.29	Literature	10.29	[47]
logP	-	3.49	Optimized	3.81	[47]
f_u	%	1.30	Literature	1.30	[56]
CYP2C19 K_M	µmol/L	83.00	Literature	83.00	[39]
CYP2C19 k_{cat}	1/min	165.23	Optimized	5.11	[39]
CYP2D6 K_M	µmol/L	2.30	Literature	2.30	[39]
CYP2D6 k_{cat} ^{EM}	1/min	37.44	Optimized	11.50	[39]
CYP2D6 k_{cat} ^{PM}	1/min	0.00	Assumed	-	[39]
GFR fraction	-	1.00	Assumed	-	-
EHC continuous fraction	-	1.00	Assumed	-	-
Partition coefficients	-	Diverse	Calculated	Be	[4]
Cellular permeabilities	-	0.32	Calculated	PK-Sim	[18]
Specific intestinal perm.	cm/min	5.23E-5	Optimized	7.23E-04	[18]

-: not given, perm.: permeability.

S3.1.2 Clinical studies

Table S3.1.2: Atomoxetine study table

Route	Dose [mg]	n	Females [%]	Age [years]	Weight [kg]	CYP2D6 activity	Dataset	References
PBPK base model building and evaluation								
po (tab, qd)	20	22	23	38 (20-49)	-	g-EM	test	Belle 2002 [3]
po (cap, sd)	40	16	33	(20-29)	(53-72)	AS = 1*	test	Cui 2007 [10]
po (cap, qd)	80	16	33	(20-29)	(53-72)	AS = 1*	test	Cui 2007 [10]
po (sol, sd)	50	42	0	23 (20-37)	62 (52-76)	g-EM	training	Nakano 2016 [33]
po (cap, sd)	50	42	0	23 (20-37)	62 (52-76)	g-EM	training	Nakano 2016 [33]
DGI model building and evaluation								
po (cap, sd)	40	18	0	23	68	AS = 0.5*	test	Byeon 2015 [7]
po (cap, sd)	40	22	0	23	65	AS = 1.25*	test	Byeon 2015 [7]
po (cap, sd)	40	22	0	23	67	AS = 2*	training	Byeon 2015 [7]
po (cap, sd)	20	8	0	(19-25)	(52-72)	AS = 0.5*	test	Kim 2018 [23]
po (cap, sd)	20	11	0	(19-25)	(49-73)	AS = 2*	training	Kim 2018 [23]
po (cap, qd)	20	3	0	35 (19-49)	-	g-PM	training	Sauer 2003 [43]
po (cap, qd)	20	4	0	45 (38-54)	-	g-EM	training	Sauer 2003 [43]
po (cap, sd)	40	12	0	(18-55)	-	g-PM	test	Todor 2016 [49]
po (cap, sd)	40	18	0	(18-55)	-	g-EM	test	Todor 2016 [49]

-: not given, *: full genotype provided in publication.

S3.2 Atomoxetine PBPK Base Model Evaluation

S3.2.1 Plasma Concentration-Time Profiles

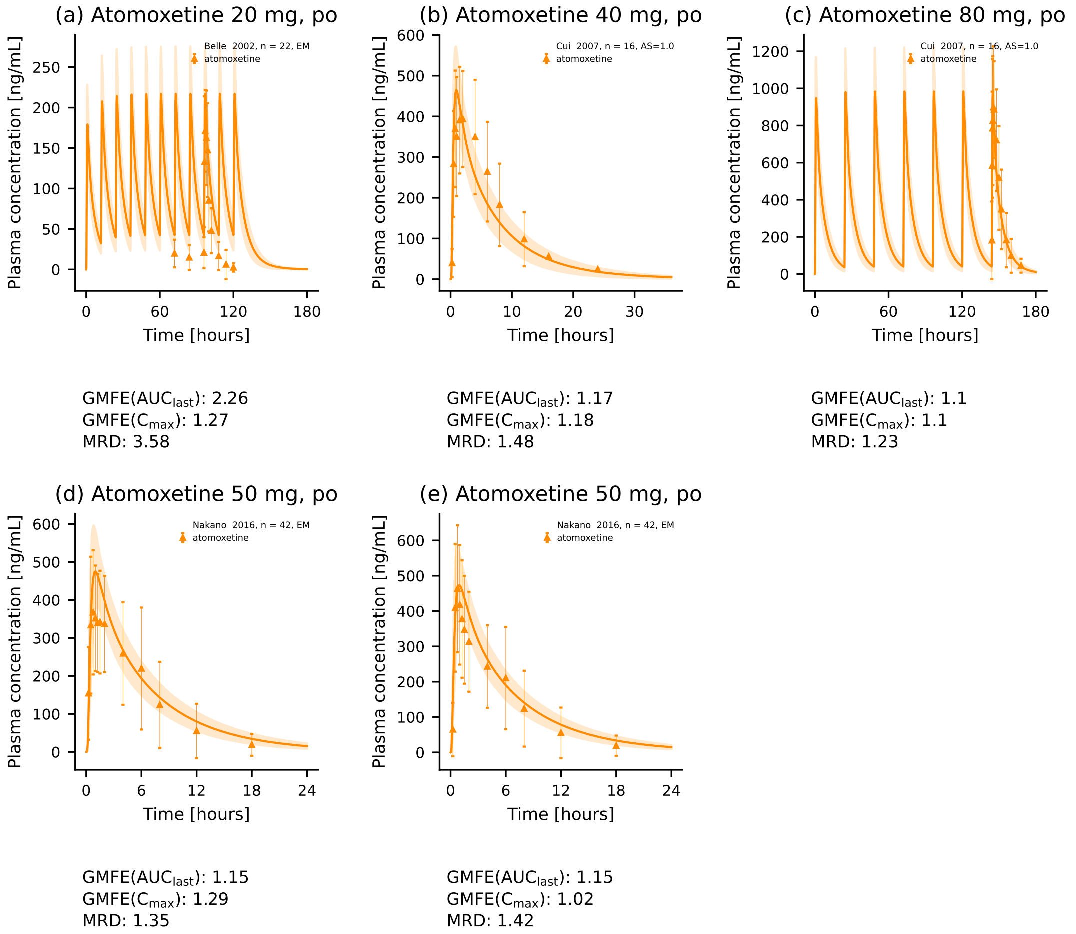

Figure S3.2.1: Atomoxetine plasma concentration-time profiles. Population predictions (n=1000) are shown as lines with ribbons (arithmetic mean ± standard deviation (SD)). Symbols represent the corresponding observed data ± SD if provided.

S3.2.2 Goodness-of-Fit Plots

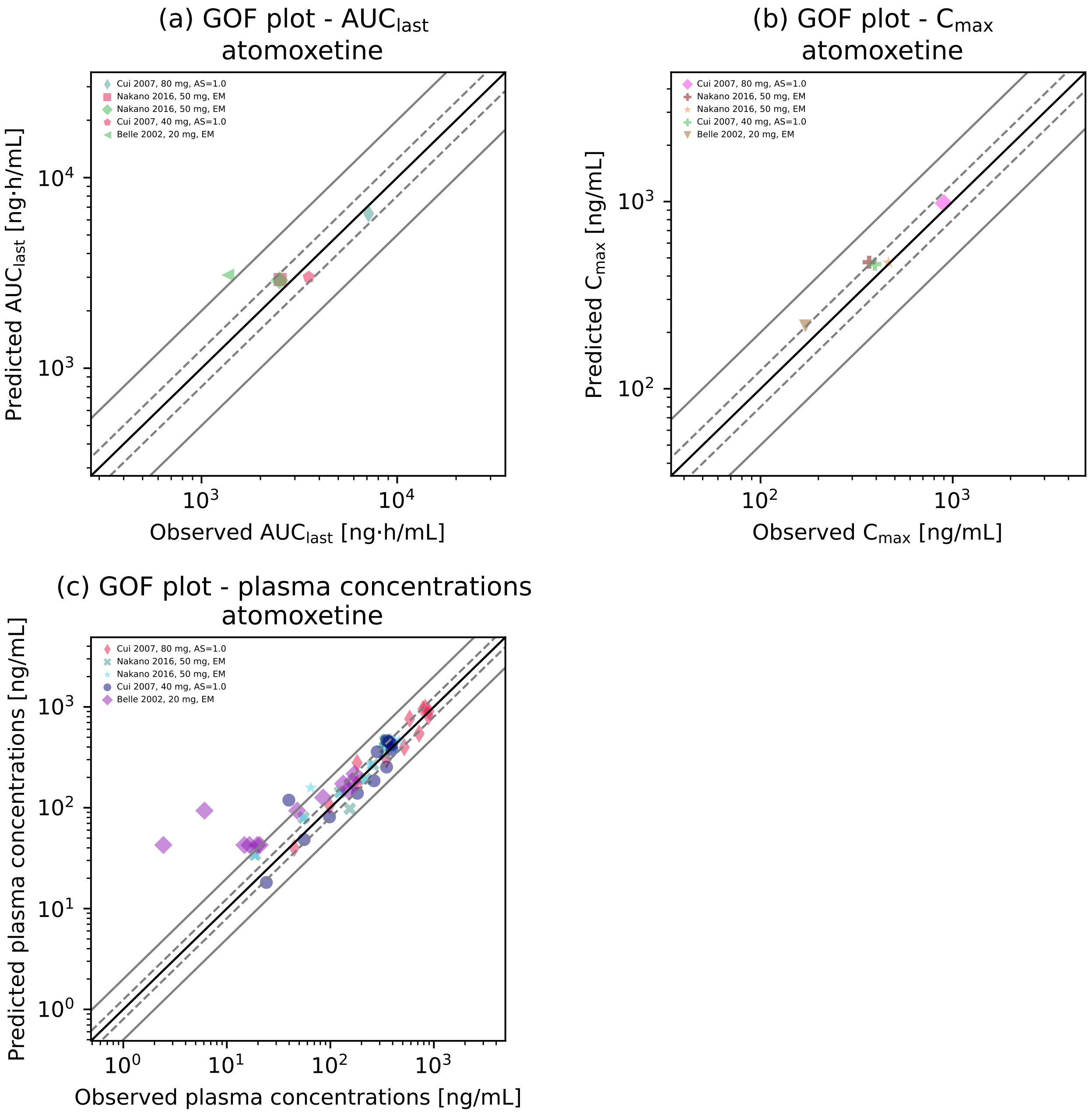

Figure S3.2.2: Goodness of fit plots. Predicted versus observed (a) AUC_{last}, (b) C_{max} and (c) plasma concentration values for all studies. The solid black line marks the line of identity, the dashed grey lines mark the 0.8- to 1.25-fold range, the solid grey lines indicate the 0.5- to 2-fold range. Colored symbols represent the study population given in the legend.

S3.2.3 Sensitivity Analysis

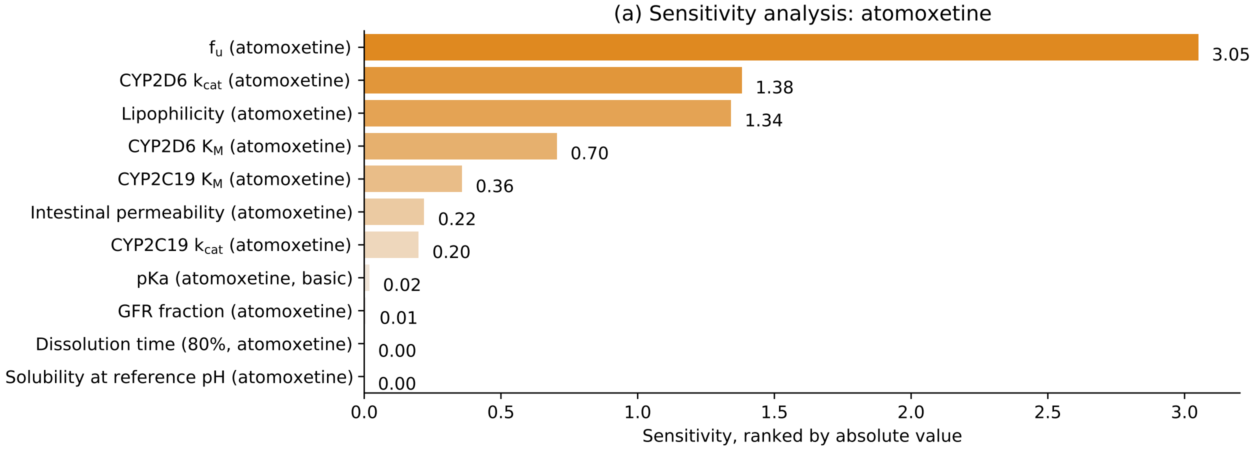

Figure S3.2.3: Sensitivity analysis of the atomoxetine model. Sensitivity of the model to single parameters, determined as change of the simulated AUC from time of the drug administration extrapolated to infinity of a single oral administration of 20 mg atomoxetine.

S3.3 Atomoxetine DGI Model Evaluation

S3.3.1 Plasma Concentration-Time Profiles

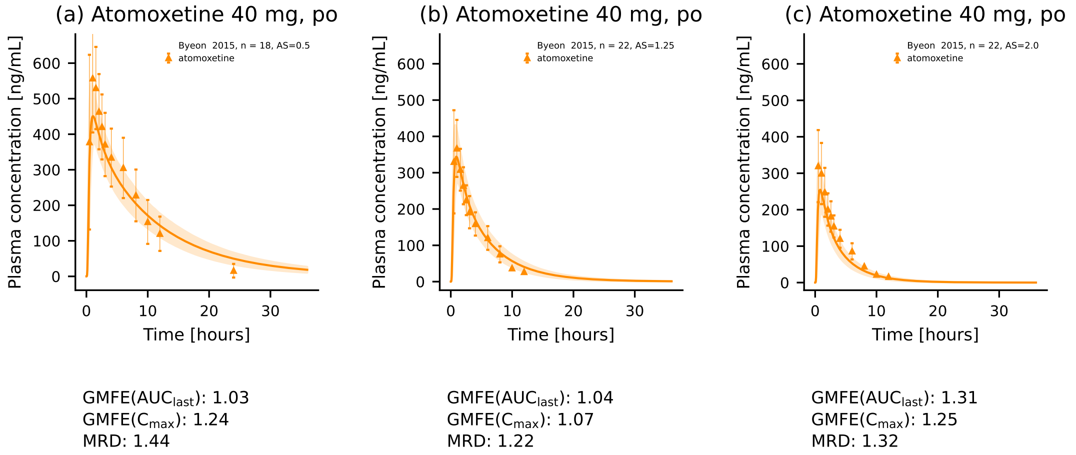

Figure S3.3.4: Atomoxetine plasma concentration-time profiles. Population predictions (n=1000) are shown as lines with ribbons (arithmetic mean ± SD). Symbols represent the corresponding observed data ± SD if provided.

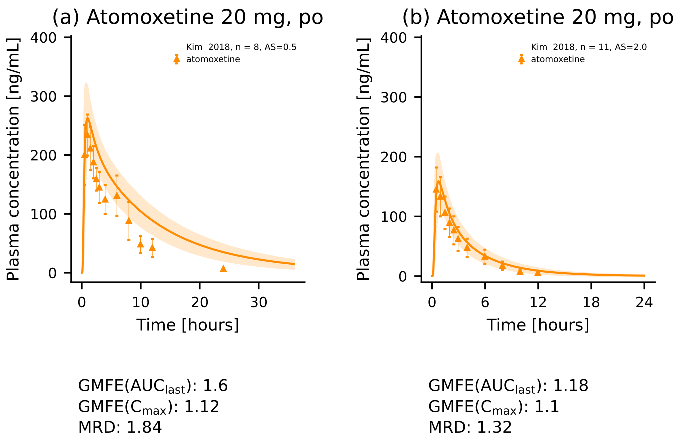

Figure S3.3.5: Atomoxetine plasma concentration-time profiles. Population predictions (n=1000) are shown as lines with ribbons (arithmetic mean ± SD). Symbols represent the corresponding observed data ± SD if provided.

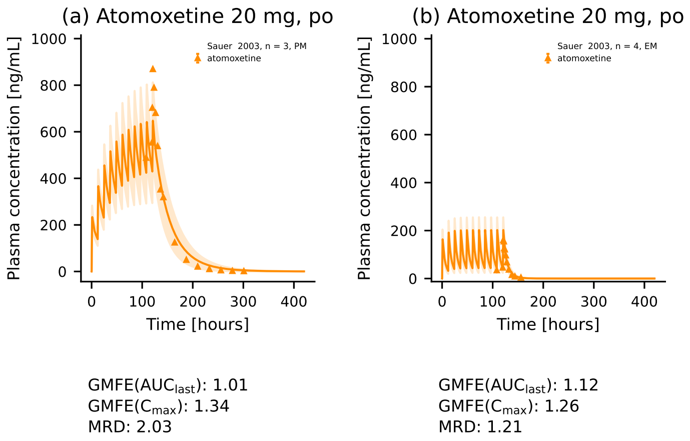

Figure S3.3.6: Atomoxetine plasma concentration-time profiles. Population predictions (n=1000) are shown as lines with ribbons (arithmetic mean ± SD). Symbols represent the corresponding observed data ± SD if provided.

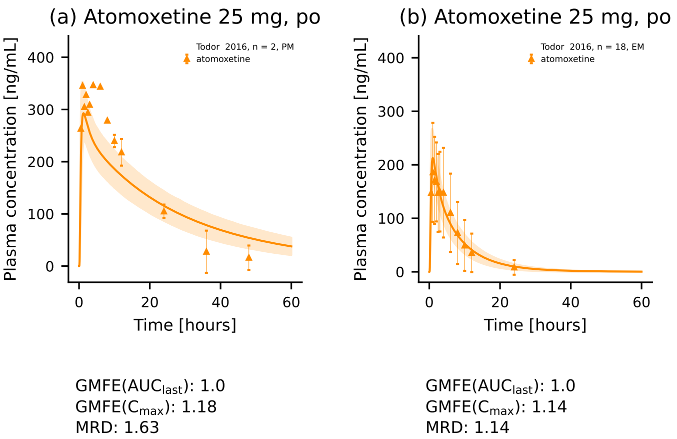

Figure S3.3.7: Atomoxetine plasma concentration-time profiles. Population predictions (n=1000) are shown as lines with ribbons (arithmetic mean ± SD). Symbols represent the corresponding observed data ± SD if provided.

S3.3.2 Goodness-of-Fit Plots

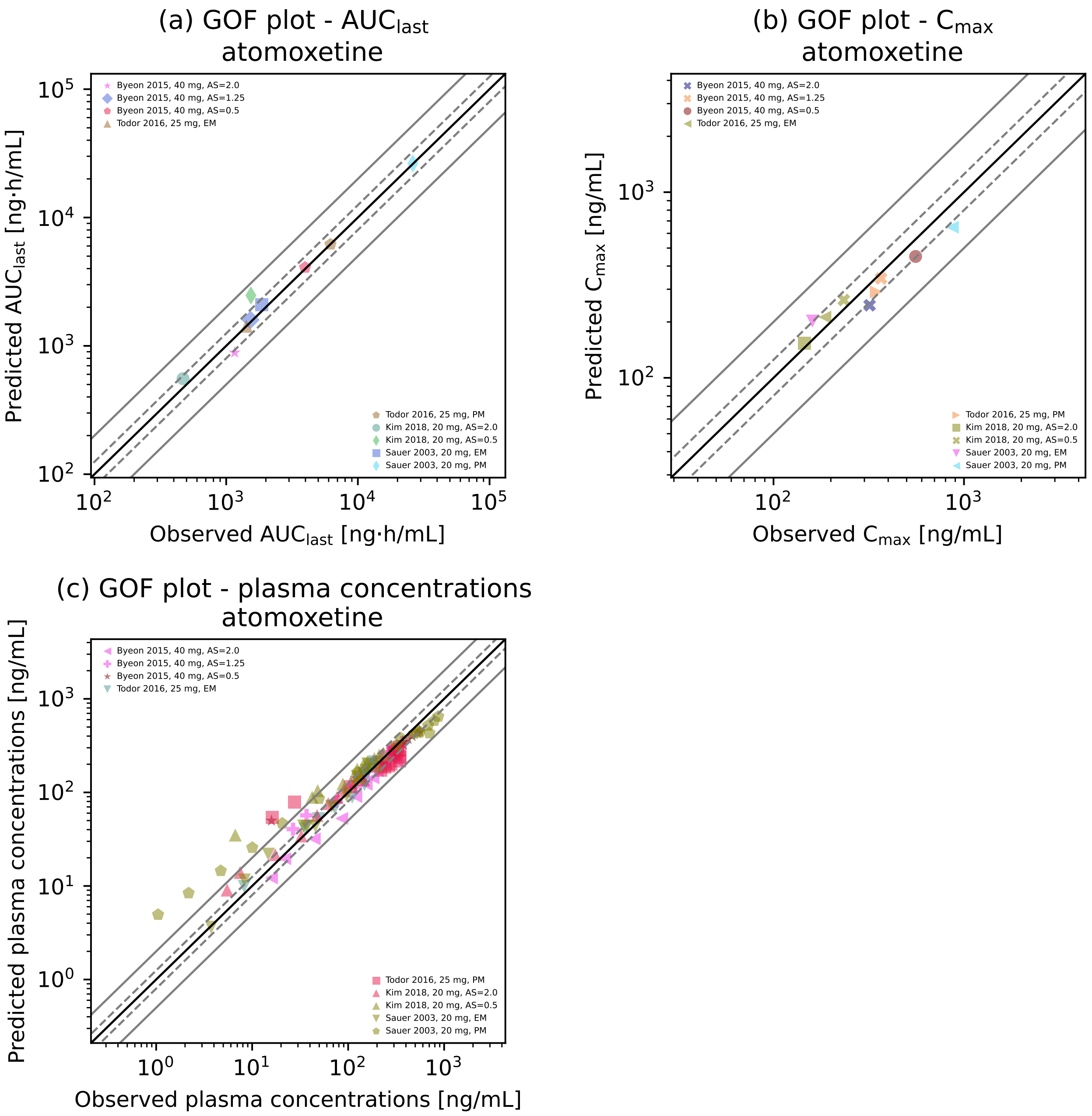

Figure S3.3.8: Goodness of fit plots. Predicted versus observed (a) AUC_{last}, (b) C_{max} and (c) plasma concentration values for all DGI studies. The solid black line marks the line of identity, the dashed grey lines mark the 0.8- to 1.25-fold range, the solid grey lines indicate the 0.5- to 2-fold range. Colored symbols represent the study population given in the legend.

S3.3.3 DGI ratios

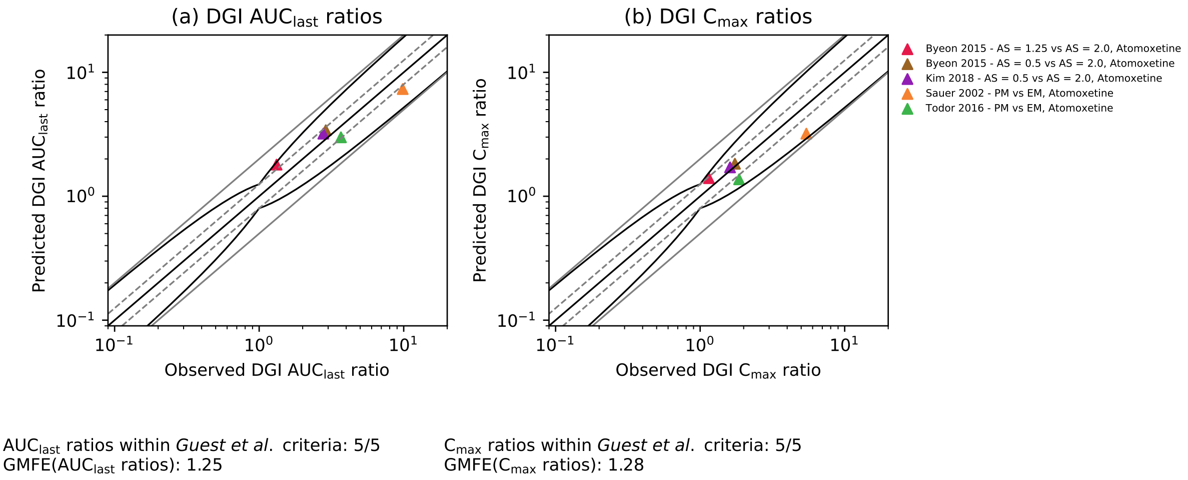

Figure S3.3.9: DGI ratio plot. Predicted versus observed DGI (a) AUC_{last} and (b) C_{max} ratios for all DGI studies. The solid straight black line marks the line of identity, the solid curved black line shows the prediction success limits proposed by *Guest et al.* [14], the dashed grey lines mark the 0.8- to 1.25-fold range, the solid grey lines indicate the 0.5- to 2-fold range. Colored symbols represent the study population given in the legend.

S4 Risperidone

For the risperidone PBPK model, a published model by Kneller et al. [24] was used. Here, most model parameters were used unchanged from the initial model. However, as the intestinal permeability for risperidone as well as system-dependent parameters were not reported in the article, and the model could not be reproduced entirely from the reported values, minor refinements were made.

S4.1 Risperidone PBPK Base Model Building

S4.1.1 Drug-dependent parameters

Table S4.1.1: Drug-dependent parameters for the final risperidone PBPK model

Parameter	Unit	Value	Source	Literature	Reference
Risperidone					
MW	g/mol	410.48	Literature	410.48	[24]
pKa (base)	-	8.76	Literature	8.76	[24]
pKa (acid)	-	3.11	Literature	3.11	[24]
Solubility (pH 7.3)	mg/mL	0.17	Literature	0.17	[24]
logP	-	2.40	Literature	2.40	[24]
f_u	%	17.50	Literature	17.50	[24]
CYP3A4 K_M → 9-HR	µmol/L	61.00	Literature	61.00	[24]
CYP3A4 k_{cat} → 9-HR	1/min	0.70	Literature	0.70	[24]
CYP3A4 K_M → sink	µmol/L	61.00	Literature	61.00	[24]
CYP3A4 k_{cat} → sink	1/min	0.15	Literature	0.15	[24]
CYP2D6 K_M → 9-HR	µmol/L	1.10	Literature	1.10	[24]
CYP2D6 ${k_{cat}}^{EM}$ → 9-HR	1/min	1.07	Optimized	2.30	[24]
CYP2D6 ${k_{cat}}^{PM}$ → 9-HR	1/min	0.00	Literature	0.00	[24]
CYP2D6 K_M → sink	µmol/L	1.10	Literature	1.10	[24]
CYP2D6 ${k_{cat}}^{EM}$ → sink	1/min	0.67	Optimized	1.40	[24]
CYP2D6 ${k_{cat}}^{PM}$ → sink	1/min	0.00	Literature	0.00	[24]
P-gp K_M	µmol/L	26.30	Literature	26.30	[24]
P-gp k_{cat}	1/min	12.72	Optimized	0.20	[24]
GFR fraction	-	1.00	Assumed	-	-
Partition coefficients	-	Diverse	Calculated	R&R	[40]
Cell permeabilities	cm/min	1.95E-03	Calculated	PK-Sim	[18]
Specific intestinal perm.	cm/min	8.04E-06	Optimized	-	[18]
9-Hydroxyrisperidone					
MW	g/mol	426.48	Literature	426.48	[24]
pKa (base)	-	8.76	Literature	8.76	[24]
pKa (acid)	-	3.11	Literature	3.11	[24]
Solubility (pH 6.5)	mg/mL	0.17	Literature	0.17	[24]

-: not available, 9-HR: 9-hydroxyrisperidone, perm.: permeabilities.

Table S4.1.1: Drug-dependent parameters for the final risperidone PBPK model

Parameter	Unit	Value	Source	Literature	Reference
logP	-	2.10	Literature	2.10	[24]
f_u	%	29.00	Literature	29.00	[24]
Unspecific CL_{hep}	1/min	0.08	Optimized	0.04	[24]
P-gp K_M	µmol/L	26.30	Literature	26.30	[24]
P-gp k_{cat}	1/min	5.70E-03	Optimized	9.64E-03	[24]
GFR fraction	-	1.00	Assumed	-	-
Partition coefficients	-	Diverse	Calculated	R&R	[40]
Cell permeabilities	cm/min	7.69E-04	Calculated	PK-Sim	[18]
Specific intestinal perm.	cm/min	3.53E-06	Calculated	3.53E-06	[18]

-: not available, 9-HR: 9-hydroxyrisperidone, perm.: permeabilities.

S4.1.2 Clinical studies

Table S4.1.2: Risperidone study table

Route	Dose [mg]	n	Females [%]	Age [years]	Weight [kg]	CYP2D6 activity	Metabolite measured	Dataset	References
PBPK base model building and evaluation									
po (tab sd)	2	36	33	32	79	-	no	training	Darwish 2015 [11]
po (tab, sd)	1	10	0	(23-38)	(65-80)	AS = 1.25*	yes	test	Kim 2008 [22]
po (tab, sd)	1	11	21	28 (22-42)	-	-	no	training	Markowitz 2002 [28]
po (tab, sd)	2	10	0	33 (23-44)	64 (55-76)	-	yes	training	Mahatthanatrakul 2012 [27]
po (tab, sd)	4	10	0	31	(55-76)	-	no	test	Mahatthanatrakul 2007 [26]
po (tab, sd)	1	12	0	24 (20-28)	65 (53-86)	AS = 1*	yes	test	Nakagami 2005 [32]
DGI model building and evaluation									
po (tab, qd)	2	8	27	43 (18-63)	-	g-EM	no	training	Bondolfi 2001 [6]
po (tab, qd)	2	3	27	43 (18-63)	-	g-PM	no	training	Bondolfi 2001 [6]
po (tab, sd)	1	6	33	24 (19-27)	67 (51-86)	AS = 0*	yes	test	Novalbos 2010 [36]
po (tab, sd)	1	26	58	23 (19-27)	65 (43-106)	AS = 1*	yes	test	Novalbos 2010 [36]
po (tab, sd)	1	33	55	23 (19-27)	66 (46-89)	AS = 2*	yes	training	Novalbos 2010 [36]
po (tab, sd)	1	6	17	23 (19-34)	73 (56-81)	AS = 3*	yes	test	Novalbos 2010 [36]

-: not given, *: full genotype provided in publication.

S4.2 Risperidone PBPK Base Model Evaluation

S4.2.1 Plasma Concentration-Time Profiles

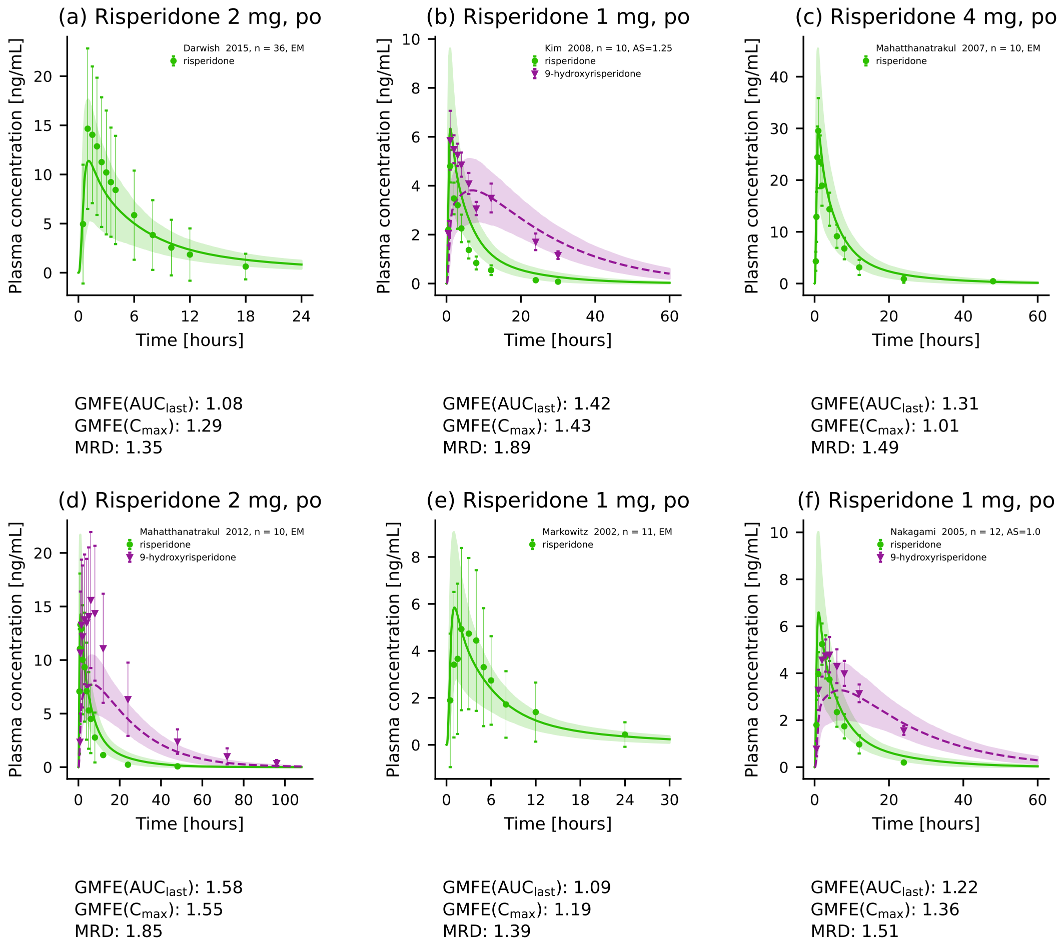

Figure S4.2.1: Risperidone and 9-hydroxyrisperidone plasma concentration-time profiles. Population predictions (n=1000) are shown as lines with ribbons (arithmetic mean ± SD) Individual predictions (n=1) are shown as lines. Symbols represent the corresponding observed data ± SD if provided.

S4.2.2 Goodness-of-Fit Plots

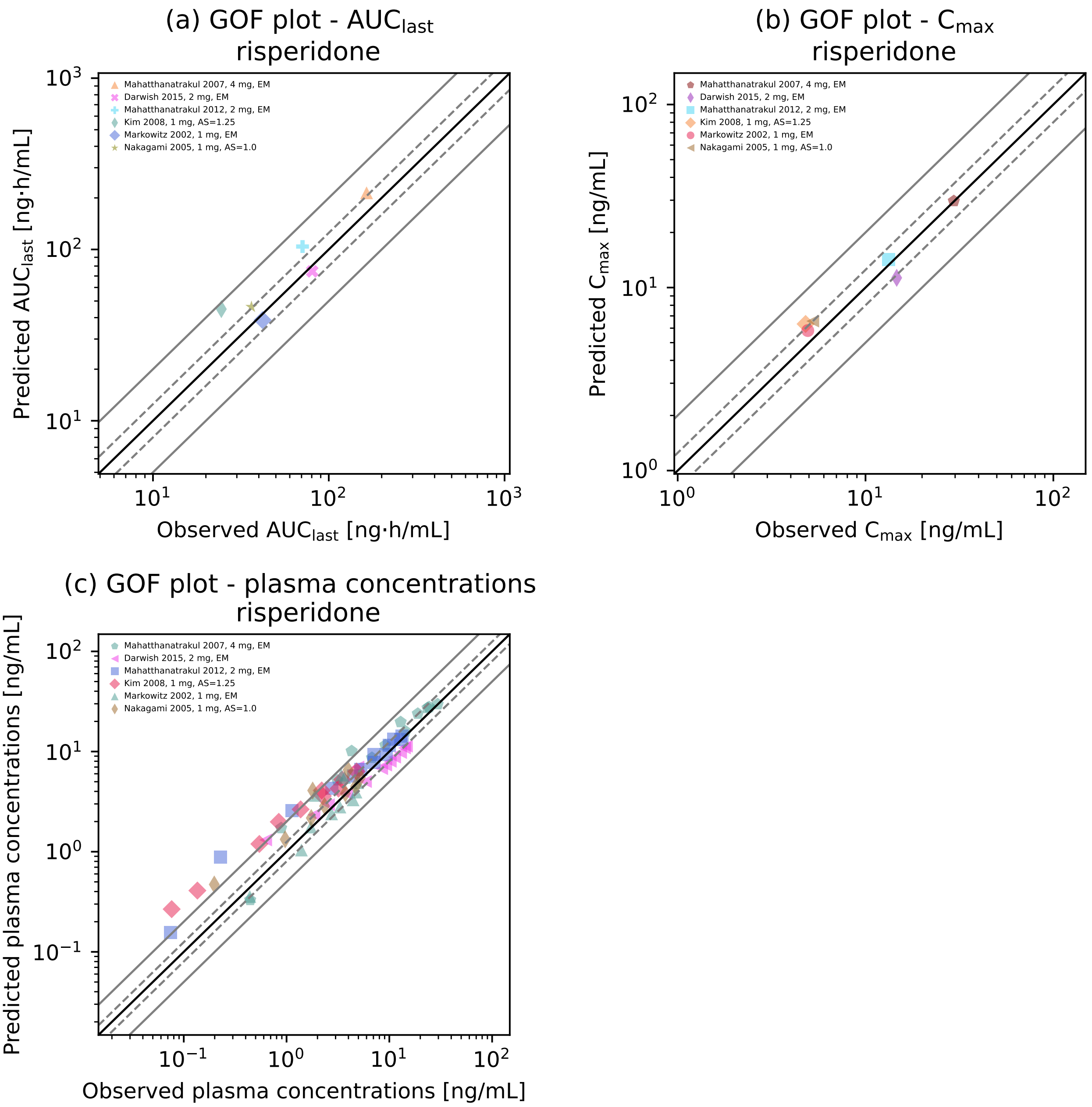

Figure S4.2.2: Goodness of fit plots. Predicted versus observed (a) AUC_{last}, (b) C_{max} and (c) plasma concentration values for all studies. The solid black line marks the line of identity, the dashed grey lines mark the 0.8- to 1.25-fold range, the solid grey lines indicate the 0.5- to 2-fold range. Colored symbols represent the study population given in the legend.

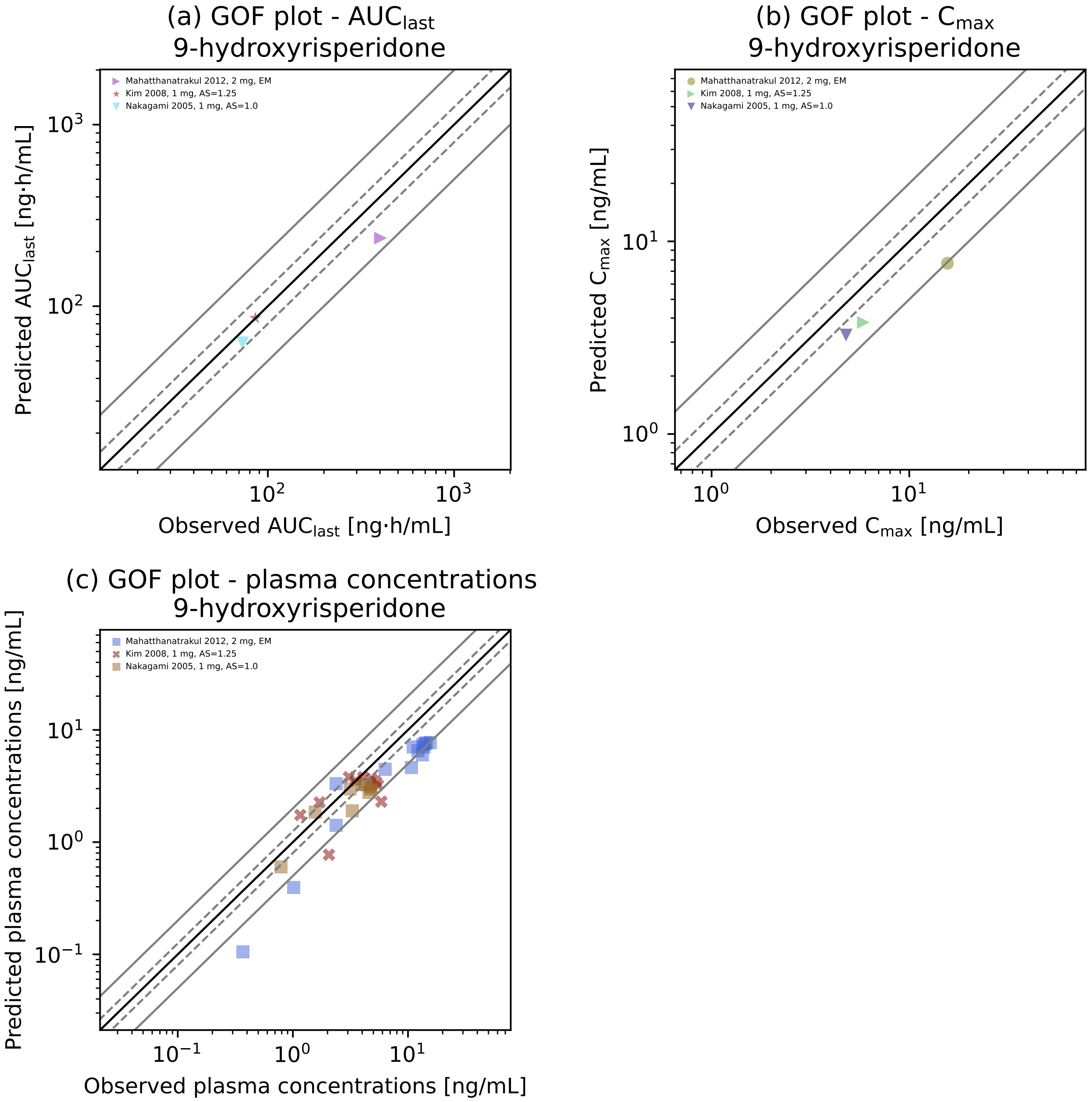

Figure S4.2.3: Goodness of fit plots. Predicted versus observed (a) AUC_{last}, (b) C_{max} and (c) plasma concentration values for all studies. The solid black line marks the line of identity, the dashed grey lines mark the 0.8- to 1.25-fold range, the solid grey lines indicate the 0.5- to 2-fold range. Colored symbols represent the study population given in the legend.

S4.2.3 Sensitivity Analysis

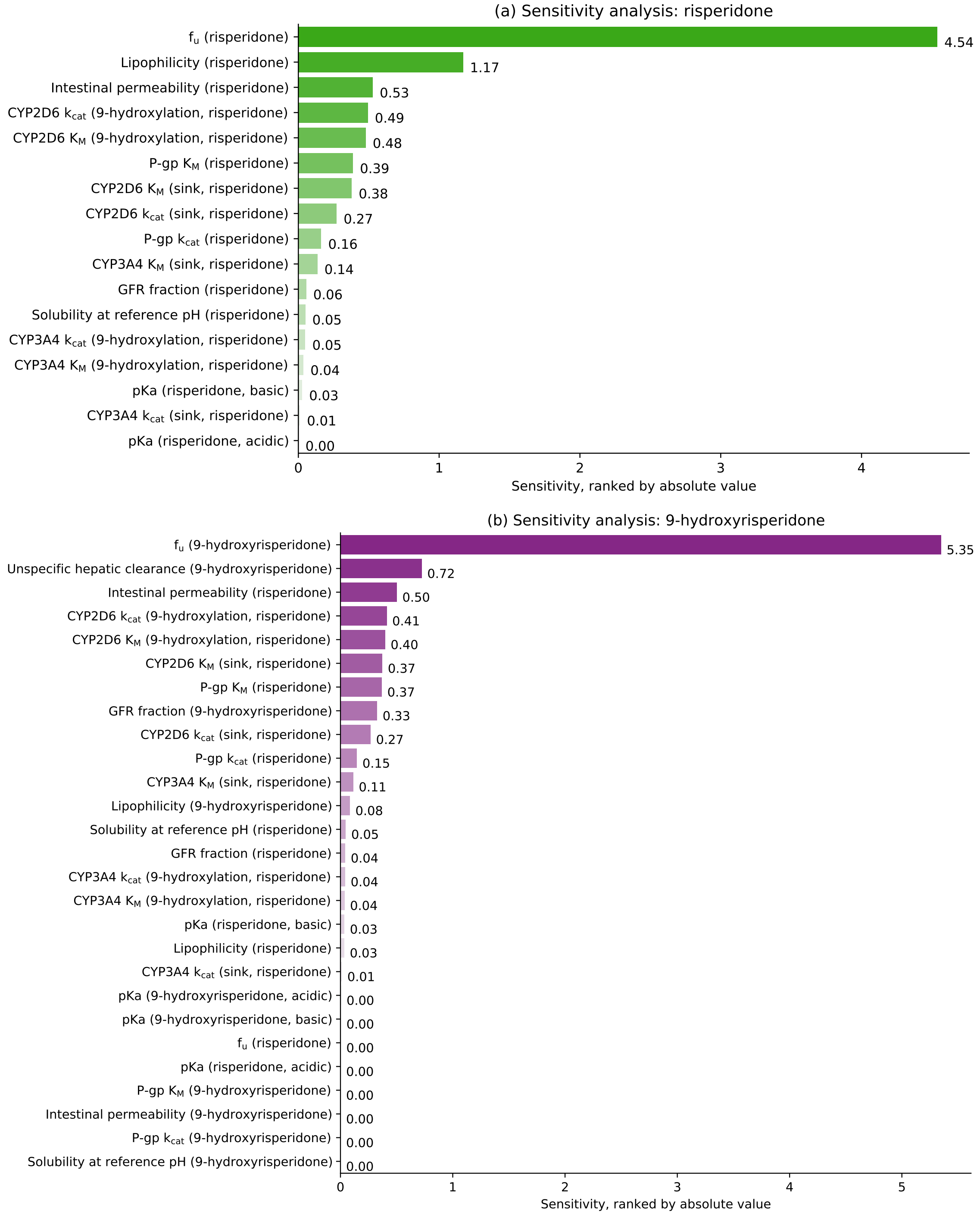

Figure S4.2.4: Sensitivity analysis of the risperidone (a) and 9-hydroxyrisperidone (b) model. Sensitivity of the model to single parameters, determined as change of the simulated AUC from time of the drug adminstration extrapolated to infinity of a single oral administration of 2 mg risperidone.

S4.3 Risperidone DGI Model Evaluation

S4.3.1 Plasma Concentration-Time Profiles

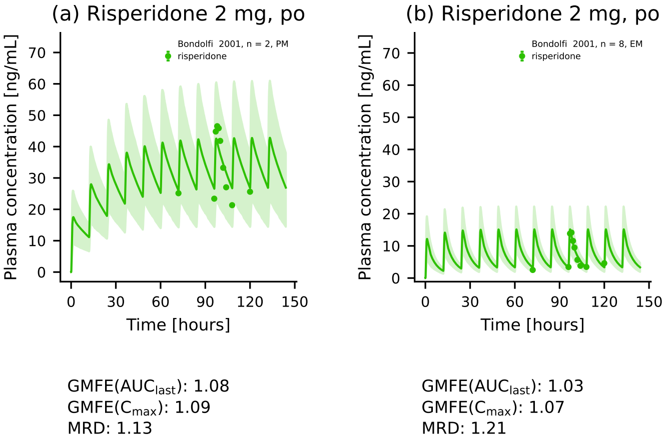

Figure S4.3.5: Risperidone plasma concentration-time profiles [9]. Population predictions (n=1000) are shown as lines with ribbons (arithmetic mean ± SD) Symbols represent the corresponding observed data ± SD if provided.

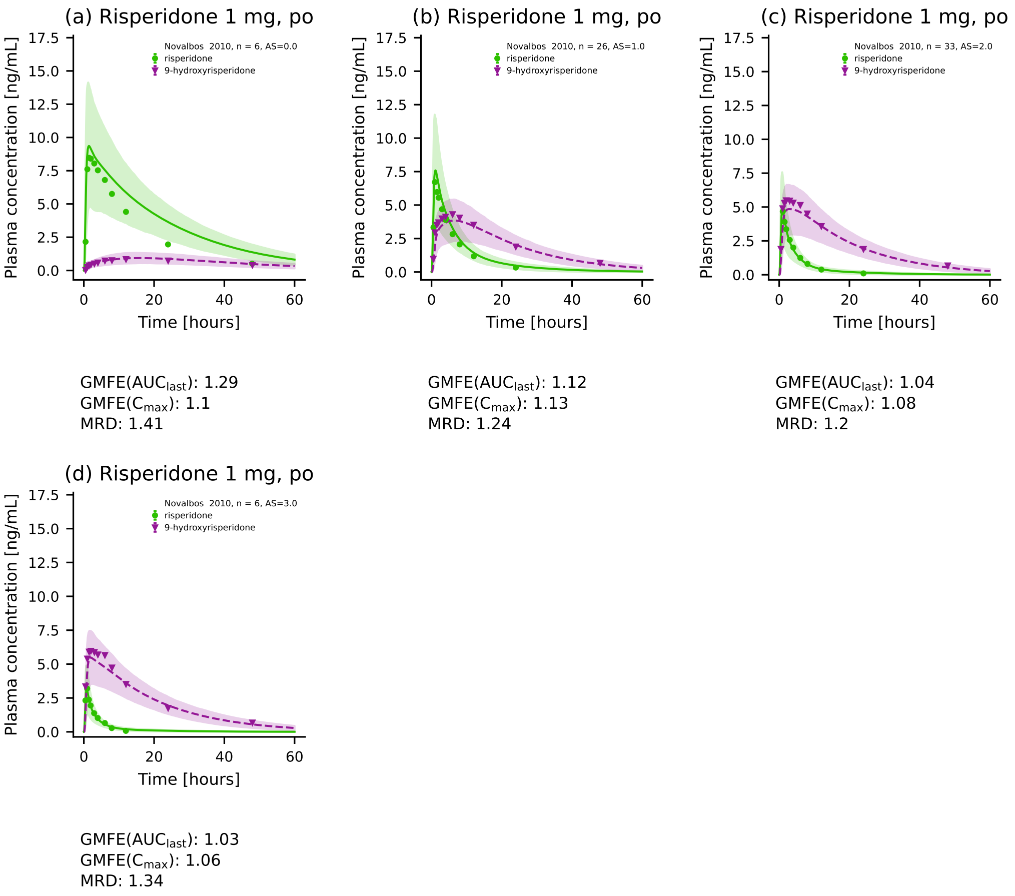

Figure S4.3.6: Risperidone plasma concentration-time profiles [12]. Population predictions (n=1000) are shown as lines with ribbons (arithmetic mean ± SD) Individual predictions (n=1) are shown as lines. Symbols represent the corresponding observed data ± SD if provided.

S4.3.2 Goodness-of-Fit Plots

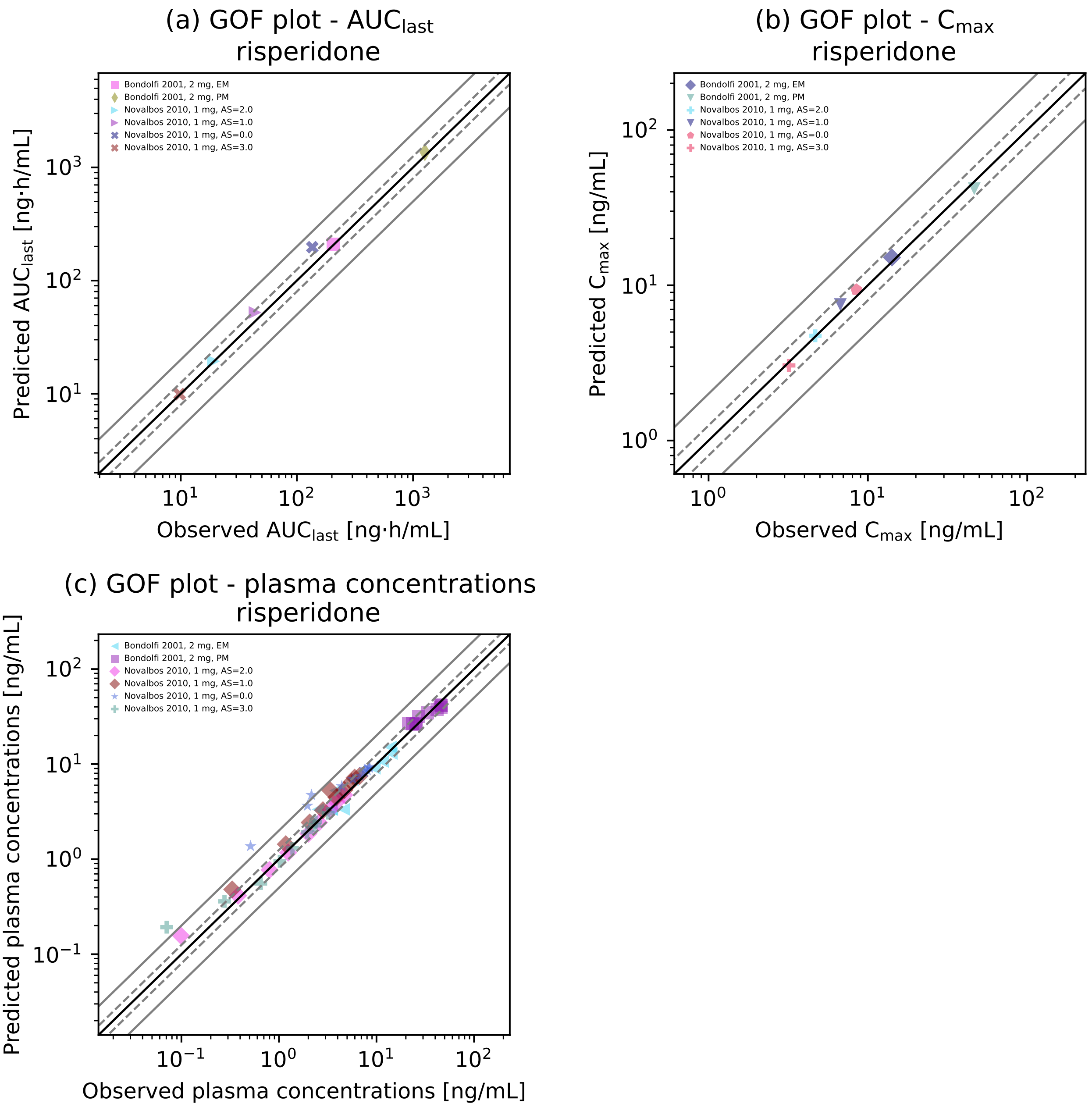

Figure S4.3.7: Goodness of fit plots. Predicted versus observed (a) AUC_{last}, (b) C_{max} and (c) plasma concentration values for all DGI studies. The solid black line marks the line of identity, the dashed grey lines mark the 0.8- to 1.25-fold range, the solid grey lines indicate the 0.5- to 2-fold range. Colored symbols represent the study population given in the legend.

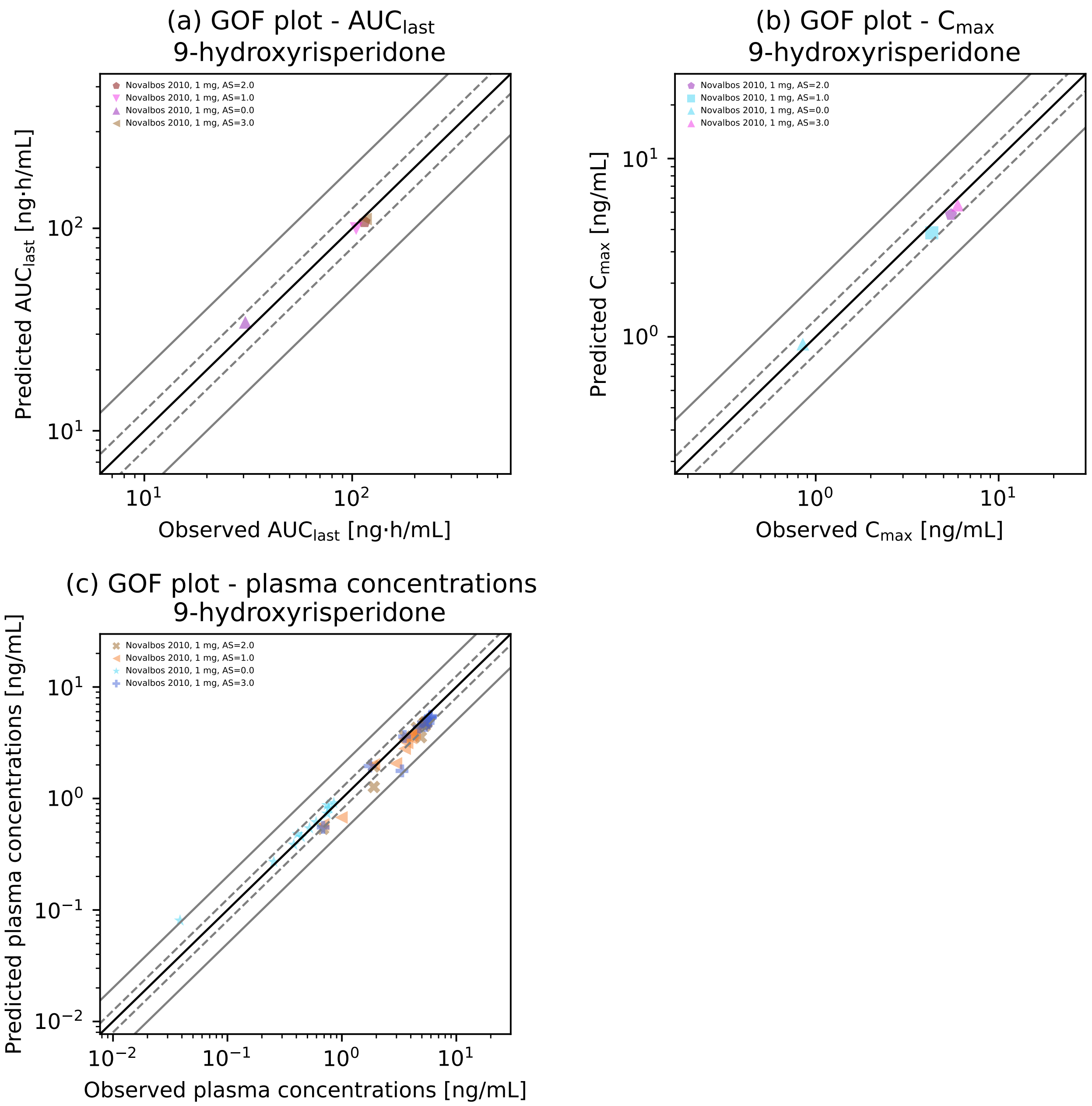

Figure S4.3.8: Goodness of fit plots. Predicted versus observed (a) AUC_{last}, (b) C_{max} and (c) plasma concentration values for all DGI studies. The solid black line marks the line of identity, the dashed grey lines mark the 0.8- to 1.25-fold range, the solid grey lines indicate the 0.5- to 2-fold range. Colored symbols represent the study population given in the legend.

S4.3.3 DGI Ratios

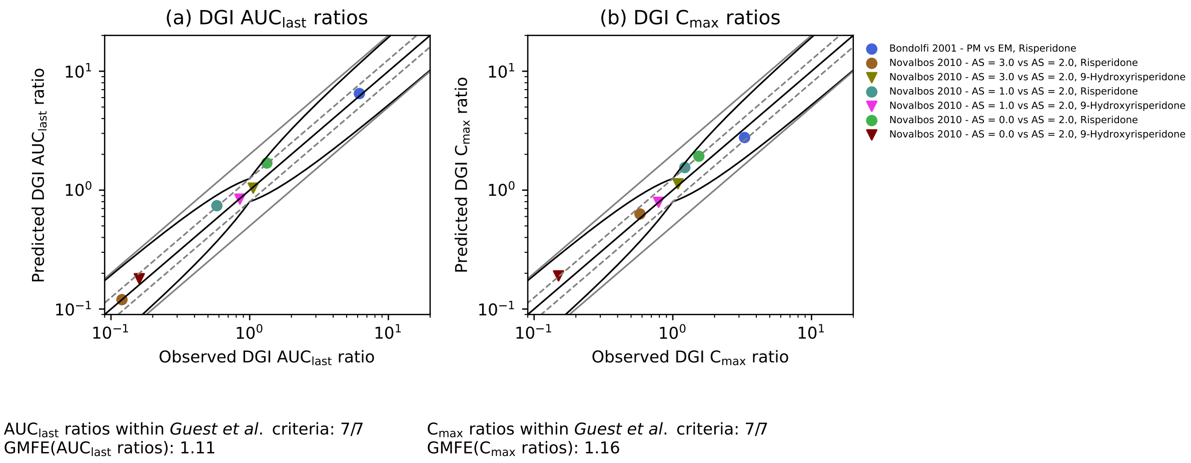

Figure S4.3.9: DGI ratio plot. Predicted versus observed (a) AUC_{last} and (b) C_{max} DGI ratios for all DGI studies. The solid straight black line marks the line of identity, the solid curved black line shows the prediction success limits proposed by *Guest et al.* [14], the dashed grey lines mark the 0.8- to 1.25-fold range, the solid grey lines indicate the 0.5- to 2-fold range. Colored symbols represent the study population given in the legend.

S5 Abbreviations

AS CYP2D6 activity score
AUC Area under the plasma concentration-time curve
AUC_{last} AUC from the time of the first concentration measurement to the last time point of concentration measurement
Be Berezhkovskiy calculation method [4]
cap Capsule
CL_{hep} Hepatic clearance
C_{max} Peak plasma concentration
CR Controlled release
CYP2C19 Cytochrome P450 2C19
CYP2D6 Cytochrome P450 2D6
CYP3A4 Cytochrome P450 3A4
DGI Drug-gene interaction
EHC Enterohepatic circulation
EM Extensive metabolizer
fu_{mic} Free fraction of compound in microsomal incubation
f_u Fraction unbound
g- Genotyped
GFR Glomerular filtration rate
ICRP International Commission on Radiological Protection
inf Infusion
iv Intravenous
k_{cat} Catalytic rate constant
K_i Dissociation constant of the inhibitor-enzyme complex
k_{inact} Maximum inactivation rate constant
K_M Michaelis-Menten constant
logP Partition coefficient
MW Molecular weight
NHANES Third National Health and Nutrition Examination Survey
p- Phenotyped
P-gp P-glycoprotein
PBPK Physiologically based pharmacokinetic
pKa Acid dissociation constant
perm. Permeability
PM Poor metabolizer
po Oral

**qd**	Once daily
**R&R**	Rodgers and Rowland calculation method [40]
**RT-PCR**	Reverse transcription polymerase chain reaction
**sd**	Single dose
**SD**	Standard deviation
**sol**	Oral solution
**tab**	Tablet

Bibliography

1. Agrawal, N. Determination of Paroxetine in Pharmaceutical Preparations Using HPLC with Electrochemical Detection. *The Open Analytical Chemistry Journal* **7,** 1–5 (2013).
2. Austin, R. P., Barton, P., Cockroft, S. L., Wenlock, M. C. & Riley, R. J. The influence of nonspecific microsomal binding on apparent intrinsic clearance, and its prediction from physicochemical properties. *Drug Metabolism and Disposition* **30,** 1497–1503 (2002).
3. Belle, D. J. *et al.* Effect of potent CYP2D6 inhibition by paroxetine on atomoxetine pharmacokinetics. *Journal of clinical pharmacology* **42,** 1219–27.
4. Berezhkovskiy, L. M. Volume of distribution at steady state for a linear pharmacokinetic system with peripheral elimination. *Journal of Pharmaceutical Sciences* **93,** 1628–1640 (2004).
5. Bertelsen, K. M., Venkatakrishnan, K., Von Moltke, L. L., Obach, R. S. & Greenblatt, D. J. Apparent mechanism-based inhibition of human CYP2D6 in vitro by paroxetine: Comparison with fluoxetine and quinidine. *Drug Metabolism and Disposition* **31,** 289–293.
6. Bondolfi, G. *et al.* The effect of fluoxetine on the pharmacokinetics and safety of risperidone in psychotic patients. *Pharmacopsychiatry* **35,** 50–56 (2002).
7. Byeon, J. Y. *et al.* Effects of the CYP2* allele on the pharmacokinetics of atomoxetine and its metabolites. *Archives of Pharmacal Research* **38,** 2083–2091 (2015).
8. Calvo, G. *et al.* Lack of pharmacologic interaction between paroxetine and alprazolam at steady state in healthy volunteers. *Journal of clinical psychopharmacology* **24,** 268–76 (June 2004).
9. Chen, R., Wang, H., Shi, J., Shen, K. & Hu, P. Cytochrome P450 2D6 genotype affects the pharmacokinetics of controlled-release paroxetine in healthy Chinese subjects: Comparison of traditional phenotype and activity score systems. *European Journal of Clinical Pharmacology* **71,** 835–841.
10. Cui, Y. M. *et al.* Atomoxetine pharmacokinetics in healthy Chinese subjects and effect of the CYP2D6*10 allele. *British Journal of Clinical Pharmacology* **64,** 445–449 (2007).
11. Darwish, M., Bond, M., Yang, R., Hellriegel, E. T. & Robertson, P. Evaluation of Potential Pharmacokinetic Drug-Drug Interaction Between Armodafinil and Risperidone in Healthy Adults. *Clinical Drug Investigation* **35,** 725–733 (2015).
12. Ganchev, B. *Charakterisierung der metabolischen Bioaktivierung des Clomifens unter besonderer Berücksichtigung genetischer Polymorphismen* PhD thesis (Tübingen, 2014), 149.
13. Greenblatt, D. J. *et al.* Time course of recovery of cytochrome P450 3A function after single doses of grapefruit juice. *Clinical Pharmacology and Therapeutics* **74,** 121–29 (2003).
14. Guest, E. J., Aarons, L., Houston, J. B., Rostami-Hodjegan, A. & Galetin, A. Critique of the two-fold measure of prediction success for ratios: application for the assessment of drug-drug interactions. *Drug metabolism and disposition: the biological fate of chemicals* **39,** 170–3 (Feb. 2011).
15. Hanke, N. *et al.* PBPK models for CYP3A4 and P-gp DDI prediction: a modeling network of rifampicin, itraconazole, clarithromycin, midazolam, alfentanil and digoxin. *CPT: pharmacometrics & systems pharmacology,* Supplementary document (Aug. 2018).
16. *Influence of Pharmacogenetic Factors, Paroxetine and Clarithromycin on Pharmacokinetics of Clomiphene - EudraCT 2009-014531-20.*

17. Jornil, J., Jensen, K. G., Larsen, F. & Linnet, K. Identification of cytochrome P450 isoforms involved in the metabolism of paroxetine and estimation of their importance for human paroxetine metabolism using a population-based simulator. *Drug Metabolism and Disposition* **38,** 376–385.

18. Kawai, R. *et al.* Physiologically based pharmacokinetic study on a cyclosporin derivative, SDZ IMM 125. *Journal of pharmacokinetics and biopharmaceutics* **22,** 327–65 (Oct. 1994).

19. Kaye, C. M. *et al.* A review of the metabolism and pharmacokinetics of paroxetine in man. *Acta Psychiatrica Scandinavica* **80,** 60–75 (1989).

20. Khatavkar, U. N., Jayaram Kumar, K. & Shimpi, S. L. Novel approaches for development of oral controlled release compositions of galantamine hydrobromide and paroxetine hydrochloride hemihydrate: A review. *International Journal of Applied Pharmaceutics* **8,** 1–6 (2016).

21. Khatavkar, U. N., Shimpi, S. L., Jayaram Kumar, K. & Deo, K. D. Development and comparative evaluation of in vitro, in vivo properties of novel controlled release compositions of paroxetine hydrochloride hemihydrate as against Geomatrix™ platform technology. *Drug Development and Industrial Pharmacy* **39,** 1175–1186.

22. Kim, K. A. *et al.* Effect of rifampin, an inducer of CYP3A and P-glycoprotein, on the pharmacokinetics of risperidone. *Journal of Clinical Pharmacology* **48,** 66–72 (2008).

23. Kim, S. H. *et al.* Physiologically based pharmacokinetic modelling of atomoxetine with regard to CYP2D6 genotypes. *Scientific Reports* **8,** 1–9 (2018).

24. Kneller, L. A., Abad-Santos, F. & Hempel, G. Physiologically Based Pharmacokinetic Modelling to Describe the Pharmacokinetics of Risperidone and 9-Hydroxyrisperidone According to Cytochrome P450 2D6 Phenotypes. *Clinical Pharmacokinetics* **59,** 51–65 (Jan. 2020).

25. Lund, J. *et al.* Paroxetine: Pharmacokinetics and Cardiovascular Effects after Oral and Intravenous Single Doses in Man. *Acta Pharmacologica et Toxicologica* **51,** 351–357.

26. Mahatthanatrakul, W., Nontaput, T., Ridtitid, W., Wongnawa, M. & Sunbhanich, M. Rifampin, a cytochrome P450 3A inducer, decreases plasma concentrations of antipsychotic risperidone in healthy volunteers. *Journal of Clinical Pharmacy and Therapeutics* **32,** 161–167 (2007).

27. Mahatthanatrakul, W. *et al.* Effect of cytochrome P450 3A4 inhibitor ketoconazole on risperidone pharmacokinetics in healthy volunteers. *Journal of Clinical Pharmacy and Therapeutics* **37,** 221–225 (2012).

28. Markowitz, J. S., DeVane, C. L., Liston, H. L., Boulton, D. W. & Risch, S. C. The effects of probenecid on the disposition of risperidone and olanzapine in healthy volunteers. *Clinical Pharmacology and Therapeutics* **71,** 30–38 (2002).

29. Massaroti, P. *et al.* Validation of a selective method for determination of paroxetine in human plasma by LC-MS/MS. *Journal of pharmacy & pharmaceutical sciences : a publication of the Canadian Society for Pharmaceutical Sciences, Societe canadienne des sciences pharmaceutiques* **8,** 340–7 (Aug. 2005).

30. McClelland, G. R. & Raptopoulos, P. EEG and blood level of the potential antidepressant paroxetine after a single oral dose to normal volunteers. *Psychopharmacology* **83,** 327–9 (1984).

31. Mürdter, T. *et al.* Impact of CYP2D6 genotype and co-medication with paroxetine and clarithromycin on clomiphene metabolism in vivo. *Abstracts of the 82nd Annual Meeting of the German Society for Experimental and Clinical Pharmacology and Toxicology (DGPT) in Naunyn-Schmiedeberg's Archives of Pharmacology* **389,** 8 (Feb. 2016).

32. Nakagami, T., Yasui-Furukori, N., Saito, M., Tateishi, T. & Kaneo, S. Effect of verapamil on pharmacokinetics and pharmacodynamics of risperidone: In vivo evidence of involvement of P-glycoprotein in risperidone disposition. *Clinical Pharmacology and Therapeutics* **78,** 43–51 (2005).

33. Nakano, M., Witcher, J., Satoi, Y. & Goto, T. Pharmacokinetic Profile and Palatability of Atomoxetine Oral Solution in Healthy Japanese Male Adults. *Clinical Drug Investigation* **36,** 903–911 (2016).
34. National Center for Health Statistics Hyattsville MD 20782. *Third National Health and Nutrition Examination Survey, (NHANES III)* tech. rep. (1997).
35. Nishimura, M. & Naito, S. Tissue-specific mRNA expression profiles of human ATP-binding cassette and solute carrier transporter superfamilies. *Drug metabolism and pharmacokinetics* **20,** 452–77 (2005).
36. Novalbos, J. *et al.* Effects of CYP2D6 genotype on the pharmacokinetics, pharmacodynamics, and safety of risperidone in healthy volunteers. *Journal of Clinical Psychopharmacology* **30,** 504–511 (2010).
37. Open Systems Pharmacology Suite Community. PK-Sim® Ontogeny Database Documentation, Version 7.3 (2018).
38. Prasad, B. *et al.* Interindividual variability in hepatic organic anion - transporting polypeptides and P-glycoprotein (ABCB1) protein expression: quantification by liquid chromatography tandem mass spectroscopy and influence of genotype, age, and sex. *Drug metabolism and disposition: the biological fate of chemicals* **42,** 78–88 (2014).
39. Ring, B. J., Gillespie, J. S., Eckstein, J. A. & Wrighton, S. A. Identification of the human cytochromes P450 responsible for atomoxetine metabolism. *Drug Metabolism and Disposition* **30,** 319–323 (2002).
40. Rodgers, T. & Rowland, M. Mechanistic approaches to volume of distribution predictions: understanding the processes. *Pharmaceutical research* **24,** 918–33 (May 2007).
41. Rodrigues, A. D. Integrated cytochrome P450 reaction phenotyping: attempting to bridge the gap between cDNA-expressed cytochromes P450 and native human liver microsomes. *Biochemical pharmacology* **57,** 465–80 (1999).
42. Rowland Yeo, K., Walsky, R. L., Jamei, M., Rostami-Hodjegan, A. & Tucker, G. T. Prediction of time-dependent CYP3A4 drug-drug interactions by physiologically based pharmacokinetic modelling: Impact of inactivation parameters and enzyme turnover. *European Journal of Pharmaceutical Sciences* **43,** 160–73 (2011).
43. Sauer, J. M. *et al.* Disposition and metabolic fate of atomoxetine hydrochloride: The role of CYP2D6 in human disposition and metabolism. *Drug Metabolism and Disposition* **31,** 98–107 (2003).
44. Schoedel, K. A., Pope, L. E. & Sellers, E. M. Randomized open-label drug-drug interaction trial of dextromethorphan/quinidine and paroxetine in healthy volunteers. *Clinical drug investigation* **32,** 157–69 (Mar. 2012).
45. Segura, M. *et al.* Contribution of cytochrome P450 2D6 to 3,4-methylenedioxymethamphetamine disposition in humans: Use of paroxetine as a metabolic inhibitor probe. *Clinical Pharmacokinetics* **44,** 649–660 (2005).
46. Sindrup, S. H. *et al.* The relationship between paroxetine and the sparteine oxidation polymorphism. *Clinical pharmacology and therapeutics* **51,** 278–87 (Mar. 1992).
47. Swain, M. chemicalize.org. *Journal of Chemical Information and Modeling* **52,** 613–615 (Feb. 2012).
48. Tanaka, G. & Kawamura, H. Anatomical and physiological characteristics for Asian reference man: male and female of different ages: Tanaka model. Division of Radioecology, National Institute of Radiological Sciences. Hitachinaka 311-12 Japan. NIRS-M-115 (1996).

49. Todor, I. *et al.* Evaluation of a potential metabolism-mediated drug-drug interaction between atomoxetine and bupropion in healthy volunteers. *Journal of Pharmacy and Pharmaceutical Sciences* **19,** 198–207 (2016).

50. Valentin, J. Basic anatomical and physiological data for use in radiological protection: reference values. A report of age- and gender-related differences in the anatomical and physiological characteristics of reference individuals. ICRP Publication 89. *Annals of the ICRP* **32,** 5–265 (2002).

51. Van der Lee, M. J. *et al.* Interaction study of the combined use of paroxetine and fosamprenavir-ritonavir in healthy subjects. *Antimicrobial agents and chemotherapy* **51,** 4098–104 (Nov. 2007).

52. Venkatakrishnan, K. & Obach, R. S. In vitro-in vivo extrapolation of CYP2D6 inactivation by paroxetine: prediction of nonstationary pharmacokinetics and drug interaction magnitude. *Drug metabolism and disposition: the biological fate of chemicals* **33,** 845–52 (June 2005).

53. Yasui-Furukori, N. *et al.* Effect of itraconazole on pharmacokinetics of paroxetine: the role of gut transporters. *Therapeutic drug monitoring* **29,** 45–8 (Feb. 2007).

54. Yasui-Furukori, N. *et al.* Terbinafine increases the plasma concentration of paroxetine after a single oral administration of paroxetine in healthy subjects. *European Journal of Clinical Pharmacology* **63,** 51–56 (2007).

55. Yoon, Y. R. *et al.* Relationship of paroxetine disposition to metoprolol metabolic ratio and CYP2D6*10 genotype of Korean subjects. *Clinical Pharmacology and Therapeutics* **67,** 567–576 (2000).

56. Yu, G., Li, G. F. & Markowitz, J. S. Atomoxetine: A Review of Its Pharmacokinetics and Pharmacogenomics Relative to Drug Disposition. *Journal of Child and Adolescent Psychopharmacology* **26,** 314–326 (2016).

57. Zhong, H., Mashinson, V., Woolman, T. & Zha, M. Understanding the Molecular Properties and Metabolism of Top Prescribed Drugs. *Current Topics in Medicinal Chemistry* **13,** 1290–1307 (2013).
